# Healing through faith: meeting a chaplain coupled with biblical readings could produce lymphocyte changes that correlate with brain activity (HEALING study)

**DOI:** 10.12688/f1000research.74504.2

**Published:** 2022-02-24

**Authors:** András Béres, Miklós Emri, Csaba Aranyi, Dániel Fajtai, Ferenc Nagy, Péter Szabó, Pál Bödecs, Edit Hörcsik, Éva Perpékné Papp, Ferenc Tomanek, Márta Kuti, Ágnes Petőfalviné, Hajnalka Kisdeákné, Gergely Bíró, Dániel Kovács, Bettina Bakos, Eszter Vinczen, Eszter Gál, Renáta Sillinger, Zoltán Szalai, Antal Szilágyi, Marianna Kiss-Merki, György Nagyéri, Judit Fodor, Tamás Németh, Erzsébet Papp, Imre Repa

**Affiliations:** 1 Kaposi Mor Hospital, Kaposvár, SOMOGY County, Hungary; 2 Pápa Reformed Theological Seminary, Pápa, Hungary; 3 Dr. József Baka, Diagnostic, Radiation Oncology, Research and Teaching Center, Kaposvár, SOMOGY County, Hungary; 4 University of Debrecen, Debrecen, Hungary; 5 Reformed Church in Hungary, Kiskunhalas, Barcs, Kaposvár, Hungary; 6 Hungarian Catholic Church, Kaposvár, Hungary; 7 SoftFlow Hungary Ltd., Pécs, Hungary

**Keywords:** faith, hospitalization, psychoneuroimmunology, theology, fMRI

## Abstract

**Background:** Faith and systems of beliefs are known to impact not only the emotional, but also the immunological state of believers in ways that we are just starting to understand. Moreover, clinical implications of previous studies are limited.

**Purpose** The aim of the “HEALING” (Hospital-based Ecumenical and Linguistic Immuno-NeuroloGic) Study was to examine immunological and neurological changes in hospitalized patients after meeting a chaplain coupled with biblical readings.

**Methods:** Hospitalized patients were pre-screened to find those who were the most in need of an intervention. A passage from the Bible was read to them during a meeting with the chaplain at the bedside (n= 20) or in the chapel (n= 18). No meeting occurred in the randomized control group (n=19). Blood samples were taken 30 minutes prior, and 60 minutes after the meeting to measure white blood cells (WBC), interferon gamma (IFN-γ), immunoglobulin M (IgM), IgA, IgG, and complement 3 (C3). A subgroup of the visited patients was subjected to functional magnetic resonance imaging (fMRI), where they were played an audiotape of readings of the same passage from the Bible (n=21).

**Results:** Lymphocyte counts increased more often after the more successful visits, but the immunological changes were not significant. Conversely, a significant (p _fwe_=0.003) correlation was revealed between changes in lymphocytes and activation of the angular gyrus (left BA39) during fMRI, a brain area involved in word recognition.

**Conclusions:** Although limited by the sample size and cohort study design, the findings suggest the depth of psycho-immunological changes could depend on the degree to which the chaplains’ main message is understood.

## Abbreviations

BA: Brodmann area

fMRI: functional magnetic resonance imaging

NK cells: natural killer cells

NSAID: nonsteroidal anti-inflammatory drug

For other abbreviations, see Table 1.

## Introduction

After Selye’s description on how stress modulates the immune system, ^1^ a wide series of studies have shown direct and complex relation between acute, ^2^^-^^5^ chronic stress, and the immune system, ^6^^-^^9^ with evidence revealing the long-term effects of early-life stress on the immune response. ^10^^-^^12^ Much less research has examined the modulating effect of positive emotions on immunity. Berk *et al.* found signs of immune stimulation among healthy adults after watching a humorous video. ^13^ Later, Bennett *et al.* showed that the extent of immunological changes related to interventions intended to elicit positive emotions, may largely depend on the way these emotions are subjectively perceived: after showing a humorous videotape to healthy women, they did not find significant change in NK-cell activity as compared with the control group, only when they included the level of cheerfulness in their calculations; this was measured by counting the number of pre-established metacommunicative signals, from just smiling to laughing out loud, that indicate the amount of mirthful laughter elicited among the participants (“Humor Response Scale”). ^14^ Studies traced down how positive experiences can trigger immunological changes in a chain reaction all the way down to the genetic code – creating” molecular signatures” related to mind-body interventions. ^15^

Most of the basic studies cited above were conducted with healthy adults, under non-clinical conditions, using non-personal tools (humorous videotapes), in the context of group sessions. The “SHoRT” study examined the immunological effect of positive emotions spurred by positive experiences of sick children being treated in a hospital. ^16^ In the “HEALING” (Hospital-based Ecumenical and Linguistic Immuno-NeuroloGic) study presented below, the authors tried to elicit a positive emotional effect through a meeting with a chaplain in a pre-selected, adult population. Previous studies only examined the long-term effects of religious life on the immune system, ^17^^-^^19^ or the relevant brain areas, ^20^^-^^22^ while neurological events have been studied in isolation from other physical changes; moreover, the concept of religious practice occurring in a hospital setting is a sensitive issue, posing many practical difficulties (“God at the bedside” ^23^). The “HEALING” study aimed to examine whether general psycho-neuro-immunological patterns could emerge from a single spiritual encounter within a clinical environment, or whether changes measured in previous studies were lost in the sea of other factors affecting the immune system.

Our trial is registered at www.ClinicalTrials.gov (Identifier: NCT04112121, Registration date: 02/10/2019).

## Methods

### Study design

The following randomized, parallel, open-labeled, controlled clinical trial study design was used:

1. Effect of biblical readings on immunological parameters
  • **Healing I. Measuring the effect of biblical readings at the bedside:** At the center of the measurement was the first meeting with the chaplain, coupled with biblical readings by the patient’s bed. With this measurement, we tried to evaluate what immunological change the “acceptance of the Word” elicits. (In Christian religion, the listening of a passage from the Bible is considered to be a possible way of meeting God. Thus, in Christian terminology, the term “Word of God” refers either to a specific biblical passage, to the Bible in general or directly to the person of God ^24^). The first blood sample was taken 30 minutes prior, the second blood sample was drawn after listening to the reading, 120 minutes after the first sample. Twenty patients were evaluated for this portion of the study.
  • **Healing II. Measuring the effect of biblical readings at the hospital chapel:** For this segment, as opposed to the previous measurement, the biblical reading took place in the hospital chapel, in small groups. Eighteen patients were evaluated in this portion of the study. The same biblical passage as the previous setting was used for the reading.
2. Effect of biblical readings on functional magnetic resonance imaging (fMRI) activity
  The patients from the previous two measurements were recruited for this measurement based on their mobility and the availability of the fMRI. During this portion of the study, the patients listened to biblical readings again; the same passage they first heard during one of the previous two measurements. The passage from the Bible was alternated with a control text, and a period of silence. We focused on whether any of the immunological or psychological parameters that appeared to change after the first listening showed any correlation with a change in fMRI activity.

**Figure 1.**
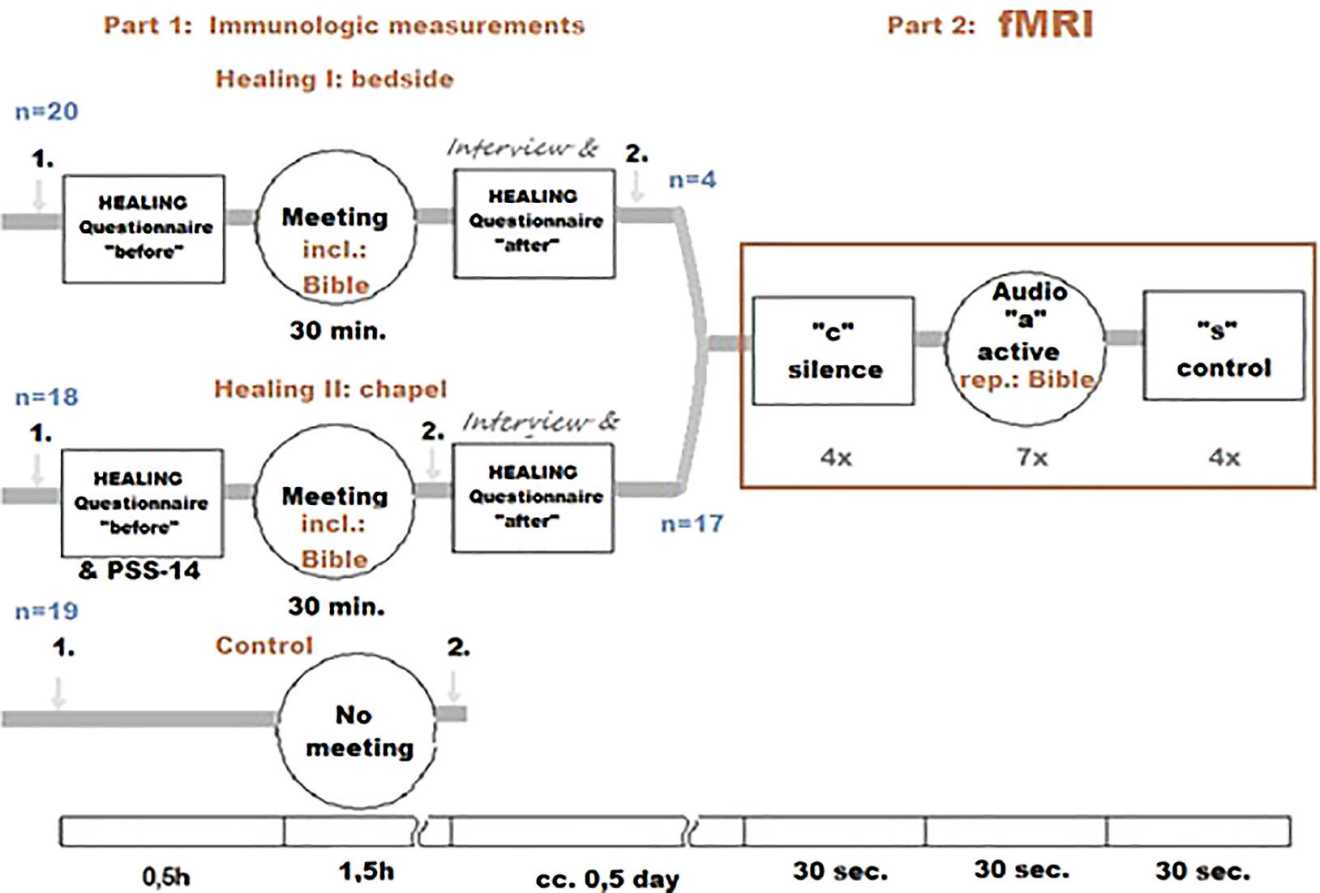
Healing Study design. “1.” and “2.”: blood samples.

For the graphic overview of the study design presented above, see Figure 1.

A total of 351 patients were screened for eligibility, 60 were randomized. Data from three patients were excluded from further analysis, because their medical condition required acute usage of oral NSAID/metamizole sodium. We analyzed results for a total of 20 patients in the bedside group (with an enrollment rate of up to two patients per week - Healing I), 18 patients in the chapel group (with an enrollment rate of three to five patients per week - Healing II), and 19 patients in the control group; we analyzed data from 57 patients in total. Of these, 22 patients were subjected to fMRI. We reported one technical failure to analyze fMRI data from one patient (the first patient in the chapel group), although the event was non-recurring. No other exclusion or loss of data occurred after randomization.

Measurements in the chapel (n = 18) occurred in five independent groups (minimum of three, maximum of five patients in each group). In three of the five groups (for 11 patients), we gave patients the option to receive communion: Catholic Eucharist, or Reformed Lord’s Supper. Of the 11 patients, five opted to receive Catholic Eucharist (four patients) or the Reformed Lord’s Supper (one patient).

The complete date range for participant recruitment was from 10 September 2015 to 2 January 2017; the nature of the interventions did not require follow up after 2 January 2017. There were no changes in trial outcome or methods after the trial commenced. The trial was stopped once the number of planned enrollments was met. We identified no harm or unintended effects on patients during or after the study.

For an overview of the enrollments, see HEALING study’s CONSORT Flow Diagram (cf. Figure 1 in the *Extended data*).

The complete protocol was approved by the Hungarian Medical Research Council’s Committee for Research Ethics (Appr.: 7245-1/2014/EKU (55./2014), authorization number: SOR/074/00130-4/2014), and our institutions’ Internal Ethical Boards (IG/02013-003/2015; 270/2015). Written informed consent was obtained from all participants. The research was conducted according to the principles expressed in the Declaration of Helsinki.

### Enrollment

Inclusion criteria were: adult age (>18 years), being hospitalized, the ability for verbal communication, alertness, orientation, no sign of psychosis in their medical history, and willingness to participate in the study after written, informed consent. The hospital’s Infectious Diseases and Nephrology Wards were involved in the recruitment process. We proposed the enrollment for all patients satisfying the above criteria, except in case of any exclusion criteria. Mobility was also an inclusion criterium for the events taking place at the chapel and the fMRI measurements. Due to the chaplain’s limited time availability, not all eligible patients were able to participate in the study. The decision was based on the patient’s degree of need and willingness, as assessed by, and at the discretion of, the chaplain, using a quick stratification scoring system specifically designed to address the practical needs of this study (see Appendix 1, *Extended data*). Exclusion criteria were the inability to communicate verbally, psychotic state (as reported by the physician responsible for the patient), altered mental state, unwillingness to participate, active and treated malignant disease, steroid, oral non-steroidal anti-inflammatory drugs (NSAIDs) or metamizole- sodium use, since these could have influenced the measured immunological parameters.

Random assignment was based on the chaplain’s availability on the day of the measurement, rather than chance allocation of all the patients willing to be visited, so that the results of the control group were not biased by disappointments or frustrations caused by the cancellation/postponement of an anticipated visit. Thus, the atmosphere in the control group reflected the genuine psychological environment of a common day at the hospital, undisturbed by out-of-the ordinary events. The randomized control group consisted of patients who knew the goal of the measurement but were explicitly asked to help with their participating in the control group, i.e., they knew they weren’t going to meet the chaplain (by request, the encounter could be scheduled for a later occasion).

Enrollment was arranged by the investigator. In order to minimize allocation bias, a covariate-adaptive, blocked, stratified randomization method was used: block size was fixed to 19 (±1) enrollments for each group, with a 1:1 allocation ratio. For the control group we enrolled patients whose diagnoses and number of days in the hospital was similar to the intervention groups, in order to ensure a good balance of participant characteristics, as the intervention groups were saturated.

### Personal encounter with the chaplain with listening to the biblical passage, and psycho-immunological measurements

**Figure 2.**
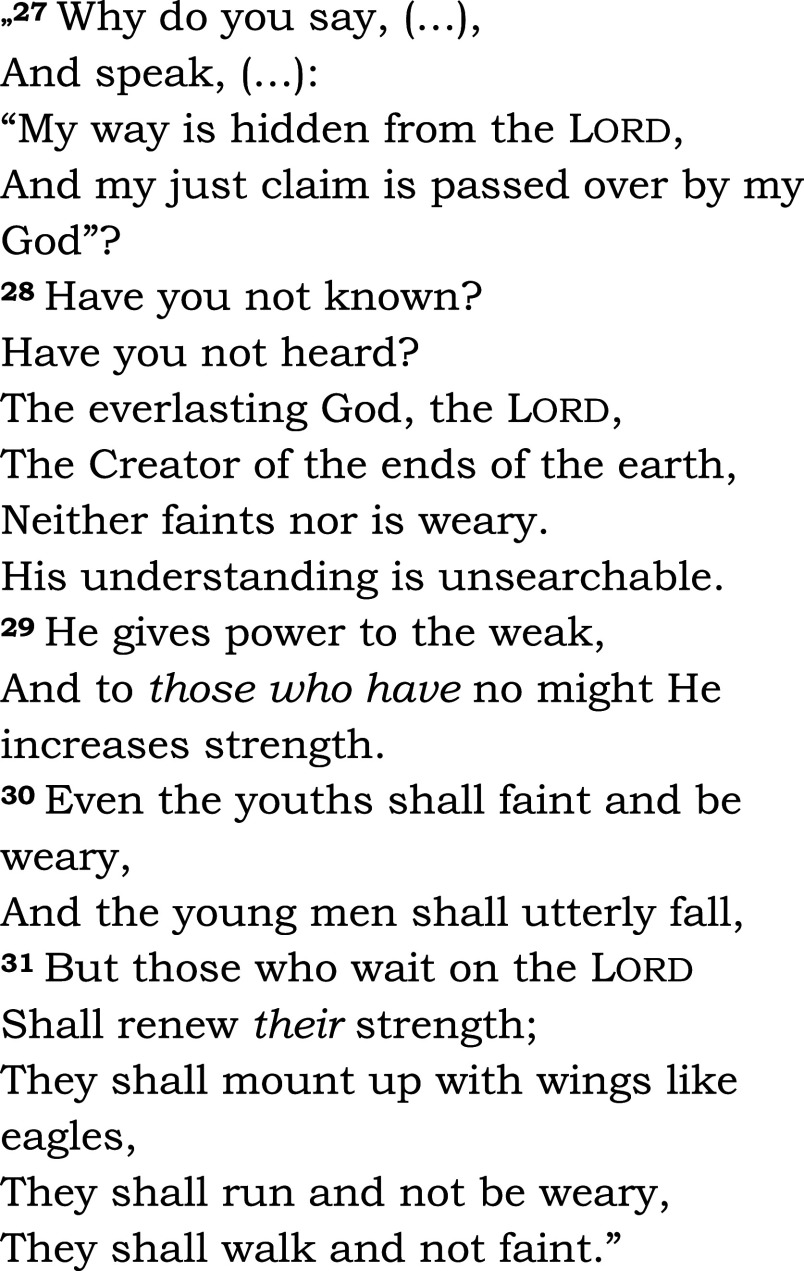
Read aloud to all patients in the bedside or chapel groups, and during subsequent fMRI-s. Isaiah 40:27-31. New King James Version (NKJV). Patients heard readings by the chaplain and in their native language, Hungarian, in the study.

The same passage was read in both groups (Isaiah 40, 27-31 – see Figure 2). Among the five groups in the chapel, communion was offered to three groups: Eucharist for Catholics, and Lord’s Supper for members of the Reformed church. We asked the patients and the chaplain to complete a questionnaire designed for the study (HEALING questionnaire, preliminary pilot testing, see Appendix 2, *Extended data*). In the groups at the chapel, we used the validated PSS-14 score. ^25^ We took blood samples 30 minutes prior to, and 60 minutes after the encounter; due to some anticipated differences in the length of the visits, which were about 30 minutes each, it was the time interval between the two samples that was fixed to 120 minutes. We supplemented the lab measurements with microscopic examination of the blood smears ^26^^-^^28^ (see Figures 4, 5, and 6), and measurements of IFNγ- (Healing I), or immunoglobulin M-, A-, G-, and C3- levels (enzyme linked immunosorbent assays), as well as some blood clotting factors (partial thromboplastin time [PTT] and international normalized ratio [INR]) (Healing II).

**Figure 3.**
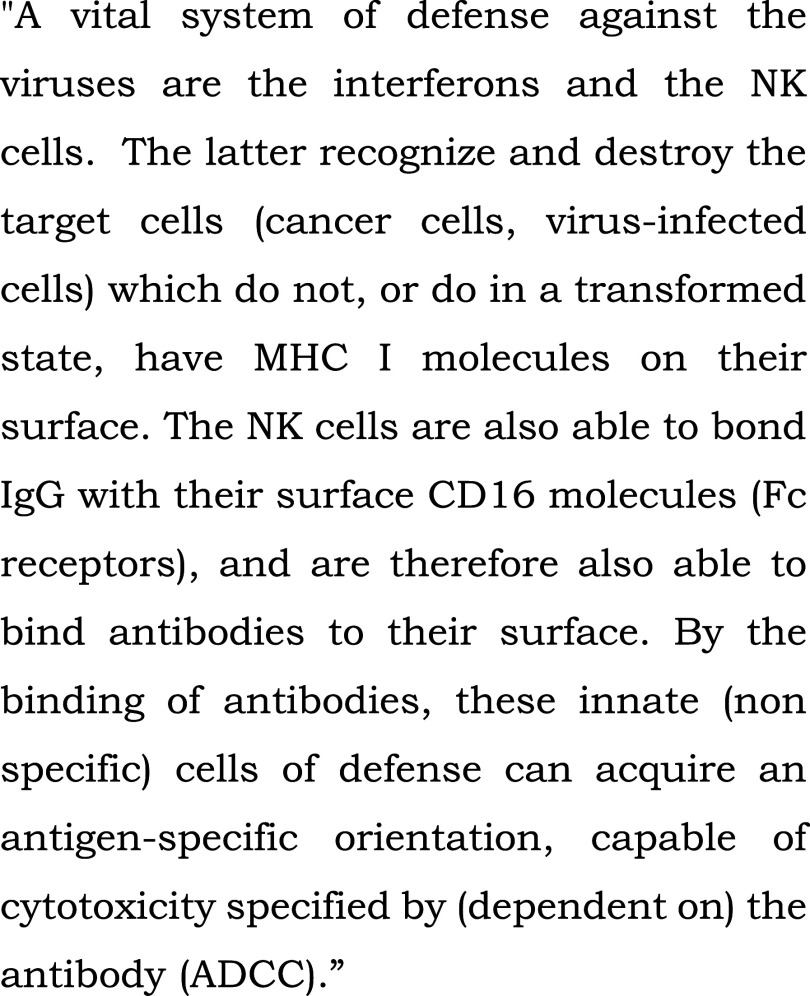
Control text read to all patients in the bedside or chapel groups during fMRI-s. “Innate (non specific) immune system” (extract). From: Szalka A, Timár L. Infektológia [Infectology]. Budapest: Medicina; 2005. Patients heard the text read by the chaplain in their native language, Hungarian, in the study.

**Figure 4.**
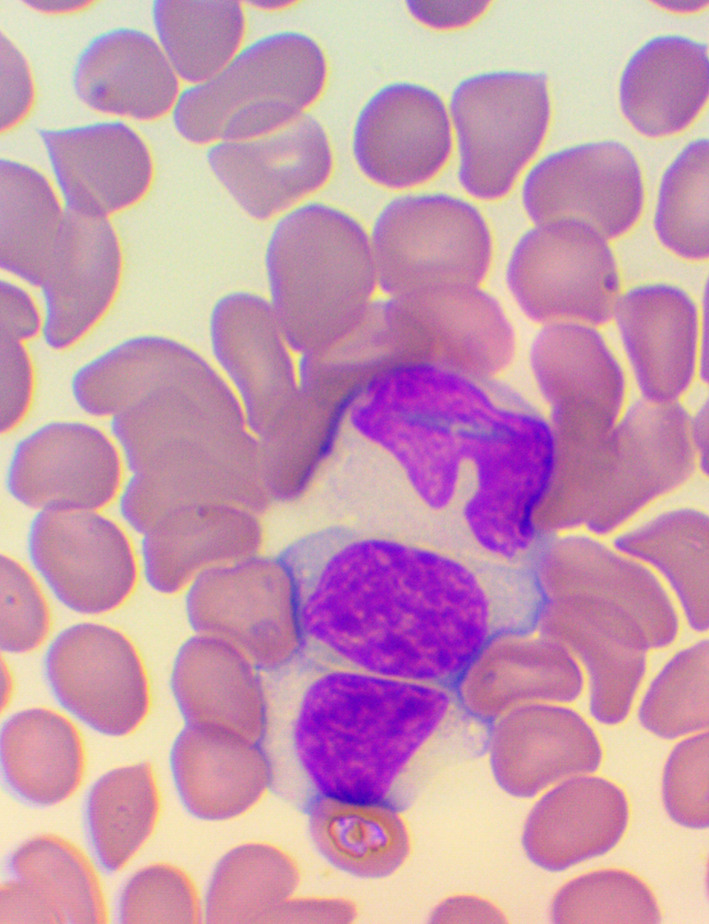
Activated lymphocytes – microscopic examination of blood smear in the Healing Study.

**Figure 5.**
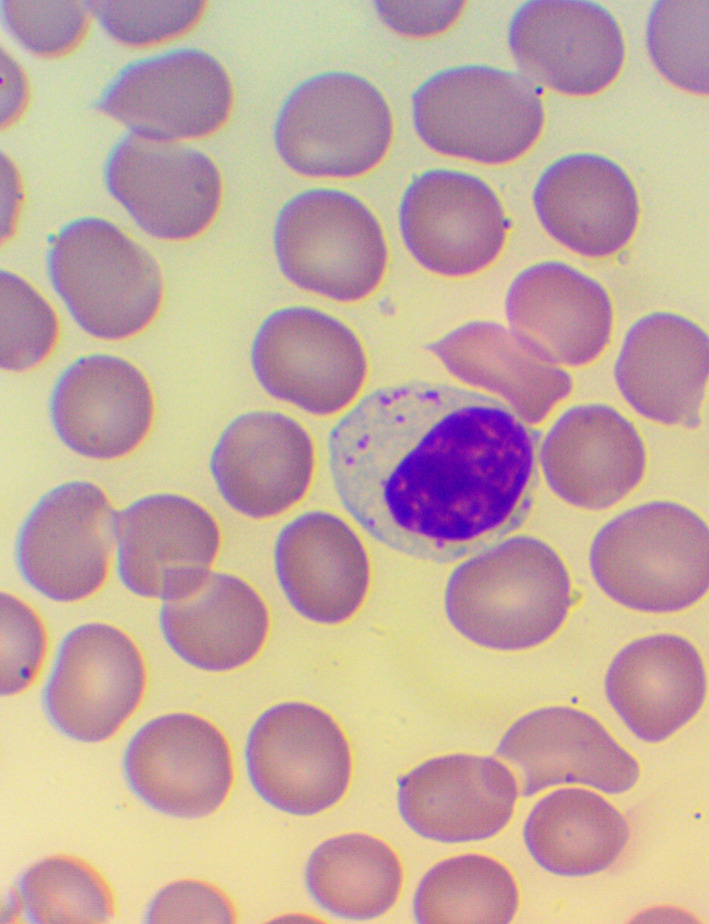
Large granuled lymphocyte (LGL) - microscopic examination of blood smear in the Healing study.

**Figure 6.**
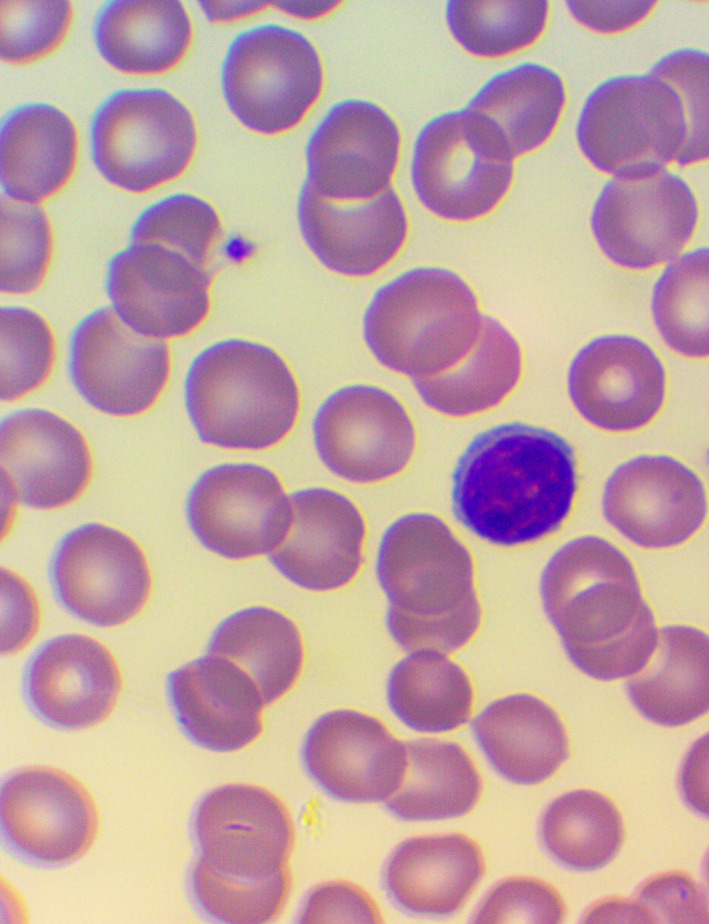
Small condensed lymphocyte - microscopic examination of blood smear in the Healing study.

For an overview of the measured psychological factors and immunological parameters, and the corresponding abbreviations, see Table 1.

**Table 1.** Psychological factors and immune-hematological parameters assessed in the Healing Study.

Psychological factors	Immune-hematological parameters
Based on the patient’s answers to the HEALING “before” questionnaire: • Patient’s personal belief on how she/he got sick (BI1 and BI2) • Patient’s desire for the visit (BII1, BII4) • Patient’s satisfaction with her/his life (BI3, BI4, BI5, BI6) • Patient’s self-assessed burden caused by the hospitalization (BI7, BIV) • Patient’s personal belief on the way she/he will be healing (BII2, BII3) • Patient’s personal faith (BII1) • Patient’s religious practice (BIII2) Perceived stress scale 14 items (PSS-14) Based on the patient’s or the chaplain’s answers to the HEALING “after” questionnaire: • Patient’s/Chaplain’s overall satisfaction with the visit (AI and PI respectively) • Patient’s assessment on the intimacy of the encounter (AII) • Chaplain’s assessment on the patient’s openness (PII) • Patient’s assessment on the sincerity of the encounter (AIII) • Chaplain’s assessment on the extent she could connect to the patient (PIII) • Patient’s assessment on the trustworthiness of the chaplain (AIV) • Patient’s/Chaplain’s assessment on the emotionally turbulent, roiling effect of the encounter (AV, PIV) • Patient’s/Chaplain’s assessment on the emotional depth of the encounter (AVI, PV) (abbreviations refer to the question number in the corresponding Healing questionnaire)	• White blood cell count (WBC) • Neutrophils: change in ✓ neutrophil count (dNeut) ✓ percentage of neutrophils (dNeut%) • Eosinophils: change in ✓ eosinophil count (dEo) ✓ percentage of eosinophils (dEo%) • Basophils: change in ✓ basophil count (dBas) ✓ percentage of basophils (dBas%) • Monocytes: change in ✓ monocyte count (dMono) ✓ percentage of monocytes (dMono%) • Lymphocytes: change in ✓ lymphocyte count (dLy-abs) ✓ percentage of lymphocytes (dLy%) ✓ percentage of large granulated lymphocytes (dLGL%) ✓ percentage of small condensed lymphocytes (dSmallLy%) ✓ percentage of activated lymphocytes (dMiddleLy%) • Lymphocyte/neutrophil ratio –change in: (dLy/Neut) • Platelet count (Plt) • Interferon-gamma level (IFNγ) • Immunoglobulin M level (IgM) • Immunoglobulin A level (IgA) • Immunoglobulin G level (IgG) • Complement C3 level (C3) • Partial thromboplastin time (PTT) • International normalized ratio (INR)

In the case of nominal values, non-parametric, associative-tests (i.e., Kolmogorov-Smirnoff) were used; in the case of immunological parameters, a normality testing followed by a parametric, paired-samples t-test was used. For all parameters measured in the study, we performed a network-analysis using two different methods: a Bayesian analyzer developed at UTE Budapest, ^29^ and R package IsingFit [R code used: IsingFit (data, family = ‘binomial’, AND = TRUE, gamma = 0.1, plot = TRUE, progressbar = TRUE, lowerbound.lambda = NA, vsize = 10)], ^30^ followed by a correlation analysis. The usually used p value of 0.05 was divided by the number of comparisons analyzed (Bonferroni correction).

### Repeated biblical readings from audiotape and fMRI measurements

Later, an fMRI examination was carried out, which was contingent upon availability of the fMRI and the capability of the patients to be mobilized. After providing written informed consent, the patients enrolled in the fMRI examination were comforted to prevent any possible anxiety related to the measuring environment (narrowness and loudness often posing a challenge for patients), given instructions on the process, then laid down into the fMRI equipment, where they could hear the reading of the study on audiotape. Patients could stop the exam at any time by pressing a button.

To examine the regions of the brain that could be impacted by the current measurement, we performed fMRI measurements using a “block-design” technique in three functional states:

1. In the active (“a”) block, patients could listen to the same passage from the Bible that they heard from their hospital bed or in the hospital’s chapel. They could hear the biblical passage once again read by the chaplain, in their native language (Hungarian) and in modern translation ( Figure 2).
2. In the control (“s”) block, the stimulus was a scientific text from audiotape, also read by the chaplain ( Figure 3). Although this text was intelligible, it contained many difficult scientific words, and complex grammatical structures in Hungarian (a Finno-Ugric language), posing an intellectual challenge for patients.
3. For a reference state, we introduced a block exposed to silence(“c”).

The MRI examinations were performed using a 1.5T Siemens Magnetom Avanto MR scanner (Syngo software versionVB17/A, Siemens Medical Solutions, Erlangen, Germany); for the timing of the stimulation and synchronization of data collection, Nordic Aktiva v1.1. equipment (Nordic Neurolab, Bergen, Norway) was used. For all patients enrolled, a structural 3D T1-weighted axial MP-RAGE recording (TE = 4.73 ms, TR = 1540 ms, TI = 800 ms, flip angle = 15°, slice-thickness 0.8 mm, 0.9 × 0.9 × 0.9 mm voxel-size) and a 3 sec. repetition time, 145 components’ blood oxygenation level dependent (BOLD) recording sequence (T2* gradient echo, TR = 3000ms, TE = 42 ms, flip angle = 90°, interleaved 4 mm axial slice thickness, 3.6 × 3.6-pixel size) was performed. During the fMRI measurements, the stimulation always started with a 15-second-long block of silence, followed by seven sections of activation blocks for 60 seconds each. The latter constituted of a 30-second-long active *,* and a 30-second-long control section or silence. During the measurements, the “block design” type stimulation was performed in a “c → as → ac → as → ac → as → ac → as” sequence order for all patients.

In the first phase of processing the fMRI image database, we assigned the T1-weighted structural images’ transformation into the MNI152 atlas-space using the FSL 5.0 and ANTS 1.9 programs ^31^^,^^32^; using the segmentation algorithm of the FreeSurfer 5.0 software package ^33^ in native space, we created the so-called brain-T1 pictures, only containing the images originating from the surface of the brain. With the help of the brain-T1 pictures, we transformed the motion-corrected fMRI picture sequences into the T1-picture corresponding to the person in question, and then with the defined atlas-space transformation we transformed it into the MNI152 atlas-space. ^31^^,^^32^ Finally, on all fMRI image sequences, after eliminating the first four recordings containing the T1-effect, we applied an 8 mm isotropic Gaussian filtering. We used the SPM12 software ^34^ to perform the statistical analysis on the created fMRI picture database at individual and population level. During the processing of the individual fMRI image sequences, using the a-c, a-s and s-c contrasts, we generated the statistical image databases (contrasting pictures) showing the differences between the effects of the various stimuli, which we used in the population-level analysis to statistically characterize the effect of the stimuli. Finally, we examined the differences in BOLD-answers linked to the active and control audio-stimuli, and their correlation with concrete clinical data corresponding to each patient. In the SPM analysis, in the comparison of the statistical differences between a-c, c-a, a-s, s-a, c-s and s-c activities, we sorted out the activation clusters containing a minimum of 100 voxels, with a Student-t = 3,58 threshold corresponding to the non-corrected p < 0.001 value from the SPM {T} pictures. We then used the MNI152 spatial coordinates of the cluster maximum, the maximal t-value, the corresponding FWE-corrected probability (peak-level inference), the size of the cluster and the FWE-corrected probability of the occurrence of the cluster (cluster-level inference) to characterize them. ^34^

## Results

The enrollment period lasted one and a half years. We analyzed 57 patients in total. The median age of the patients was 64 (Healing I), 65 (Healing II), and 66 years old (control). The only criteria when enrolling patients for the control group was that the patient’s age, type of disease, and days of treatment did not show considerable difference compared with the intervention groups; due to the small number of patients willing to participate, statistical stratification needed to be used with constraints (cf. Table 1, *Extended data*).

### Part 1: Psycho-immunological changes measured after the experience of the meeting

To the question of whether they believe in God, the majority of patients (Healing I: 65%, Healing II: 77.8%) answered positively. Only 25%-38.9% of the patients responded that they actively practiced their faith. A total of 35% and 44.4% of the patients reported cathartic, or a very positive experience at the end of the measurement (maximal rating of 5/5 at Question “AI” in the HEALING “after” questionnaire, i.e., the patient’s overall satisfaction with the visit, as reported on a single item rating scale). 60% and 66.7% reported the visits to be deeply emotional (rating of minimum 4/5 at Question “AVI” in the HEALING “after” questionnaire, i.e., the patient’s assessment on the emotional depth of the encounter, as reported on a single item rating scale).

**Figure 7.**
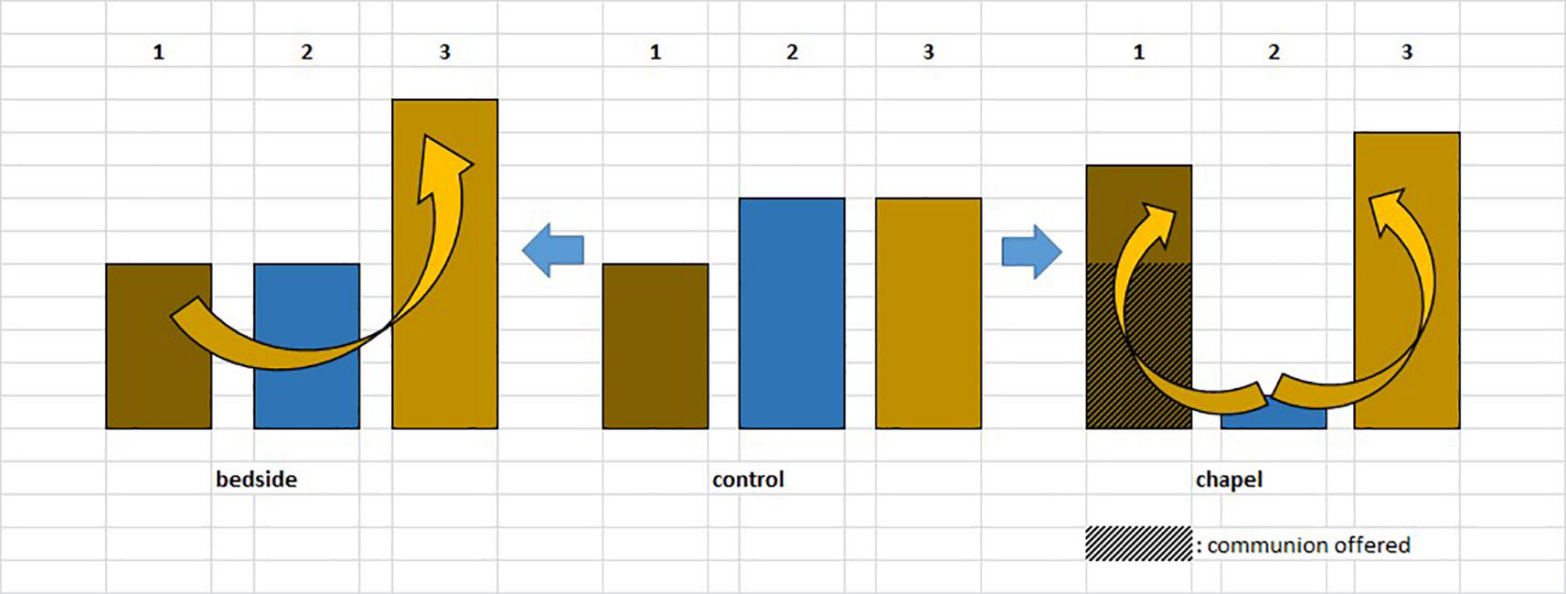
Changes in lymphocyte counts in Healing I (bedside) and Healing II (chapel) study. Columns show the number of patients. 1: lymphocyte count (Ly-abs) and percentage (Ly %) decreased; 2: Ly-abs and Ly % did not change in the same direction; 3: Ly-abs and Ly % increased.

**Figure 8.**
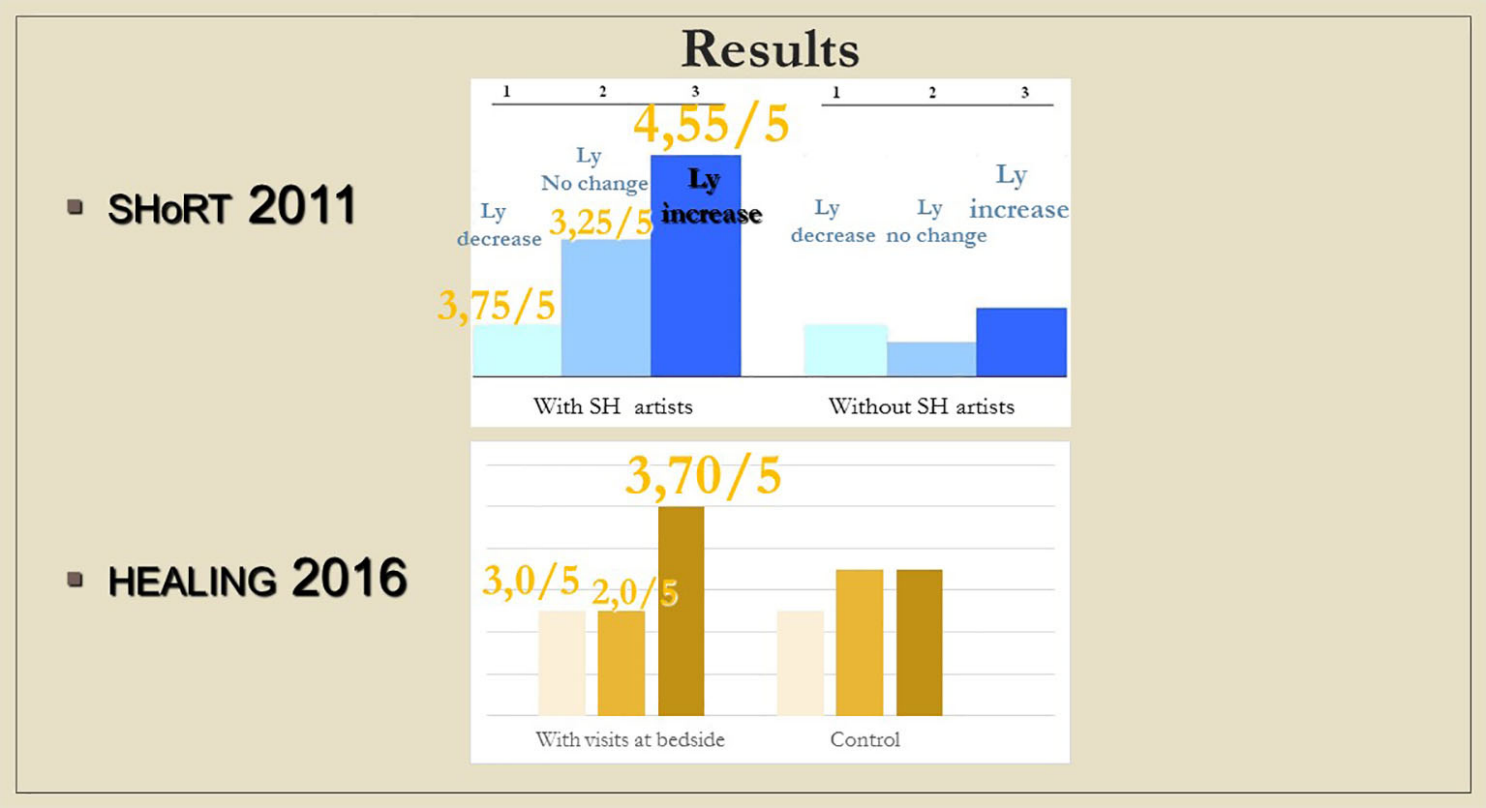
Columns show the number of patients with lymphocyte count decrease, no change and increase, respectively; in case a meeting took place, numbers above the columns indicate the average of subjective scores (from 1 to 5) by which the artists (SHoRT) or the chaplain (HEALING) evaluated the encounter.

**Figure 9.**
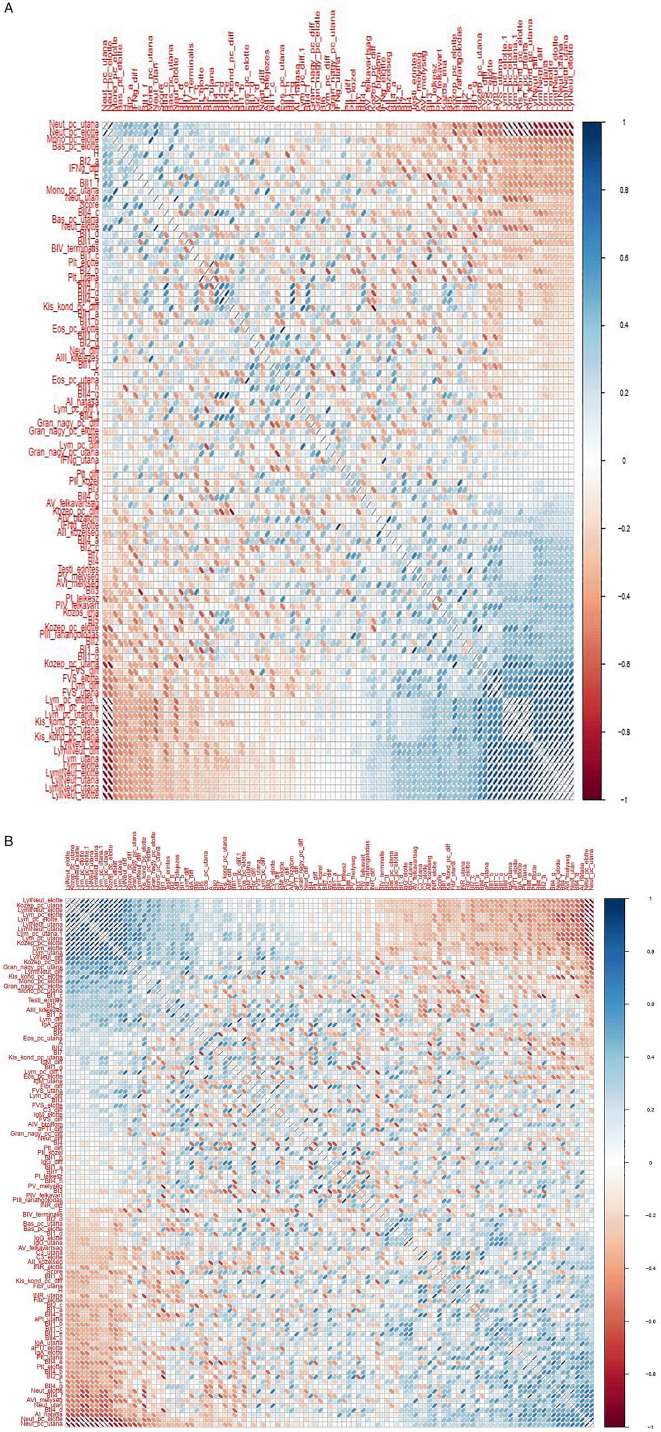
Matrix showing all possible correlations measured between psychological factors and immunological parameters, with darker colors corresponding to stronger correlations. (A) Correlation analyses – Healing I; (B) Correlation analyzes – Healing II.

**Figure 10.**
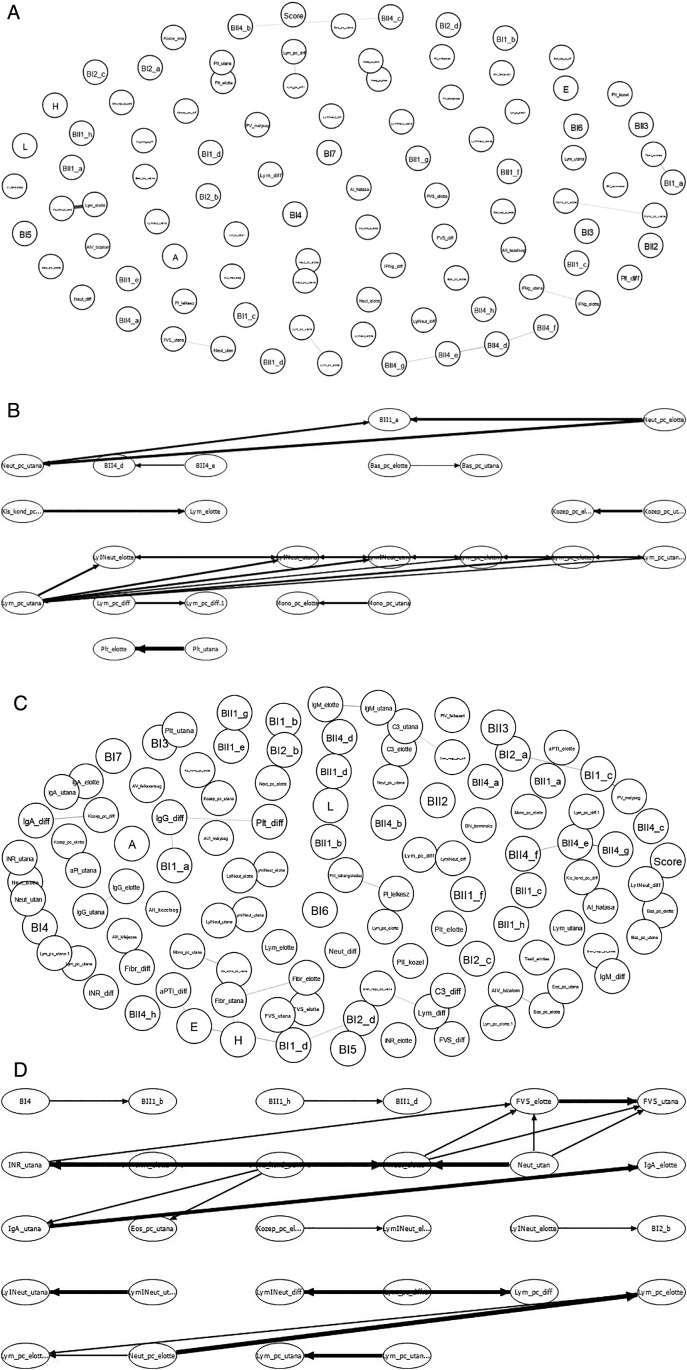
Schematic showing the examination of possible correlations between psychological factors (upper part of the chart in IsingFit) and immunological parameters (lower part of the chart in IsingFit), with thicker arrows indicating stronger correlations. (A) Analysis of correlations in Healing I with IsingFit. (B) Highlight of correlations in Healing I with a self-developed Bayesian analyzer (UTE Budapest). (C) Analyzis of correlations in Healing II with IsingFit. (D) Highlight of correlations in Healing II with a self-developed Bayesian analyzer (UTE Budapest). It appears the only - and weak - correlation between any psychological factor to any immunological parameter is the answer”b” to question”BI2” (i.e. answer “It is my fault that I became sick. I blame myself …” to the question on whether the patient feels responsible for his chronic illness (es)), correlating with neutrophile counts. Figures 9- 10: Hungarian abbreviations were used. FVS: white blood cells. Lym: lymphocytes. Neut: neutrophils. Eos: eosinophils. Bas: basophils. Mono: monocytes. LymNeut: lymphocyte-neutrophil ratio. Gran_nagy: large granulated lymphocytes (LGL). Kozep: middle-sized lymphocytes. Kis_kond: small condensed lymphocytes. pc: percentage. Plt: thrombocytes. IFNg: interferon gamma. Ig: immunoglobulin. INR: International Normalized Ratio. aPTI: partial thromboplastin time. Fibr: fibrinogen. “elotte”: before, “utana”: after. UTE Budapest = Budapest University of Technology and Economics.

Although changes in immunological parameters appeared to show tendentious deviations in both groups (bedside and chapel, see Figures 7 and 8), after Bonferroni correction, these changes were not statistically significant. Similarly, we did not find significant changes in immunological changes in the control group, therefore we reported no considerable difference between the intervention and control groups either (changes in IFNγ-levels were not considered to be clinically relevant, because the variance of the values at baseline was too elevated between the control and the intervention groups; we reported a miscalculation error retrospectively identified among PSS-14 scores – they were subsequently corrected in the raw data) (see Tables 2, 3, and 4, *Extended data*). With the statistical tools we used, we did not find significant correlation between the immunological and the psychological parameters (see Figures 9- 10). It is an interesting observation that for the patients who were willing to take part of the communion offered, we measured decrease in lymphocyte counts without exception (see Figure 7).

### Part 2: Results of the fMRI measurement

We obtained evaluable functional Magnetic Resonance Images (fMRIs) from a total of 21 patients in the second part of the study.

The comparison of the “biblical reading” block with the “silence” block showed significant (p < 0.001) activation in the right- and left BA 22 (Wernicke) and right- and left BA41 (Primary Auditory) areas solely *.* The comparison of the “control” block with the “silence” block showed significant (p < 0.001) activation in the same areas solely.

After that, we performed several subgroup analyses to explore the correlations between the psychological factors or the immunological parameters, and the changes in fMRI activities between the “active” and the “control” blocks. We created the subgroups of patients according to the extent of the change that was measured in the psychological factors (chaplain’s overall satisfaction with the visit – “PI” – and patient’s overall satisfaction with the visit and on the emotional depth of the encounter – “AI”, “AVI” - as reported on the single item rating scales corresponding to the questions of the HEALING “after” questionnaire), or the immunological parameters (changes in the lymphocyte count [dLy-abs], percentage of lymphocytes [dLy%], and lymphocyte/neutrophil ratio [dLy/Neut], values as measured automated laboratory, changes in the percentage of large granulated lymphocytes [dLGL%] as measured with microscopic examination of blood smears). The subgroups contained approximatively similar (half-half) number of patients.

It was solely among the dLy-abs subgroups that there showed to be a tendency in regards to the change in fMRI activity. We found a weak difference between activity in the area of leftBA39 between the subgroup of patients who showed an increase in lymphocyte counts, versus the subgroup of patients who showed no relevant increase in lymphocyte counts (p = 0.393). No other psychologic or immunologic pair of subgroups showed any difference in regards of their change in fMRI activity.

**Figure 11.**
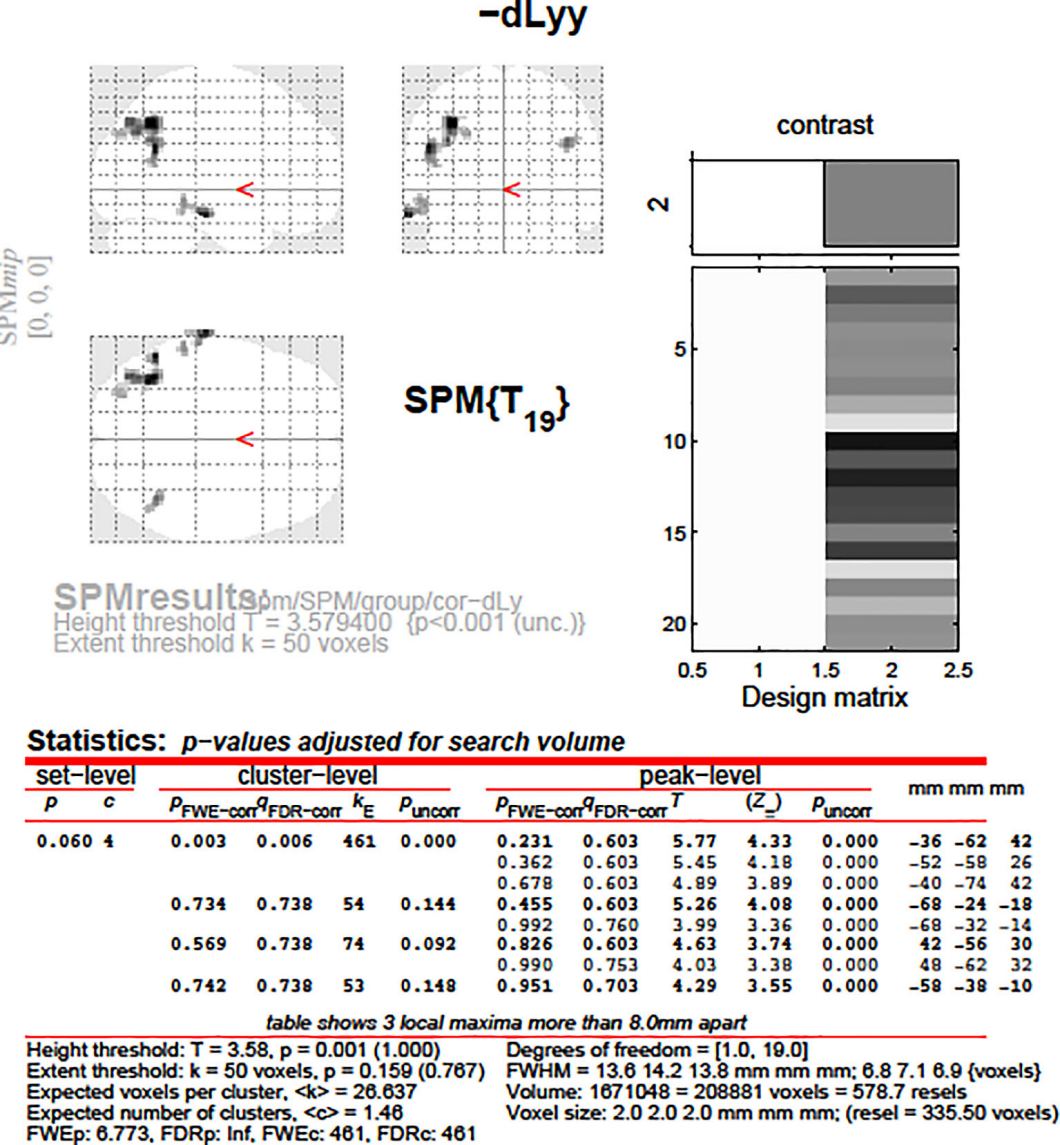
dLy-abs vs. a-s fMRI at *leftBA39*. Change in lymphocyte count (“dLy”) versus difference between activation during the active “a” and the control “s” block (“response”) during fMRI at the leftBA39 *.*

**Figure 12.**
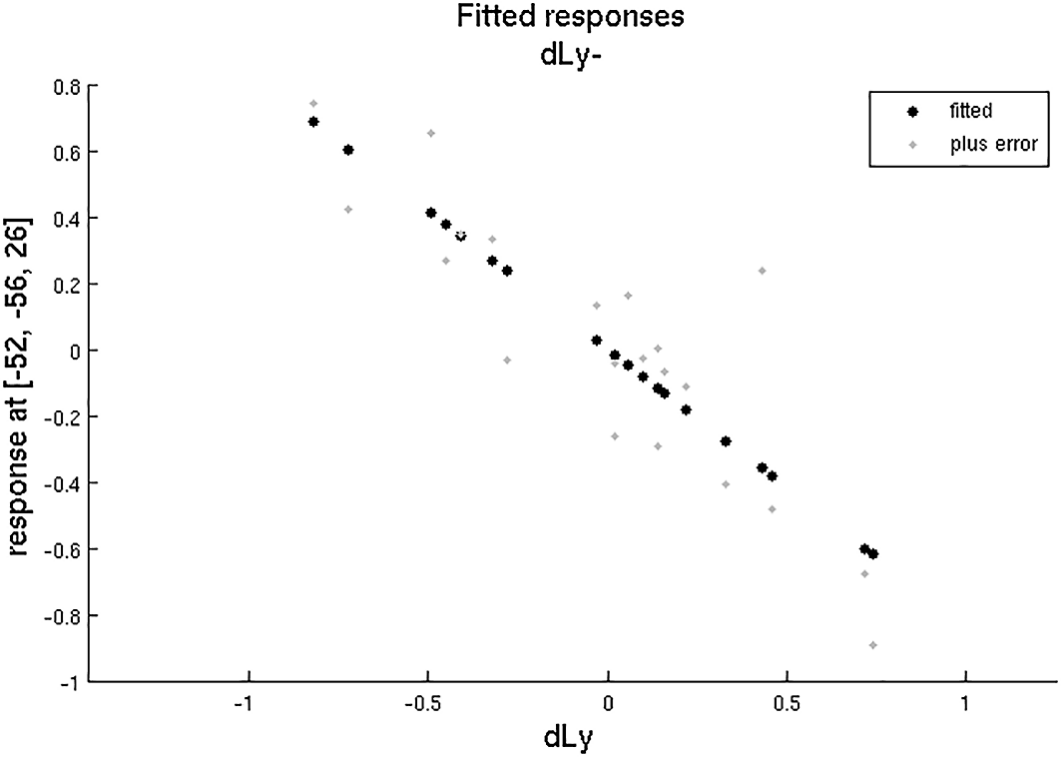
dLy vs. fMRI at *leftBA39*. Change in lymphocyte count (“dLy”) versus difference between activation during the active “a” and the control “s” block (“response”) during fMRI at the leftBA39 *.*

After that, we aimed to correlate the dLy parameters with the areas showing change in the fMRI activity. An invert linear correlation emerged (p = 0.019 with dLy%, p = 0.003 with dLy-abs) between the change in activity of the left BA39 area and the change in lymphocyte counts (see Figures 11- 12). Finally, we performed the correlation analysis between the change in fMRI activity between the “active”- “control” periods, and the dNeut, dLy/Neut, AI, AVI, and PI factors or parameters, but we found no significant correlation.

Accounting for both the correlation analyses and the subgroup analyses, altogether we examined only 12 factors or parameters, and therefore the level of significance was 0.05/12 = 0.00417. The correlation between the left BA39 area and change in lymphocyte count remained significant after the Bonferroni correction.

## Discussion

Taking Salopek’s “slow journalism” concept as a model [he makes a 21,000 mile-long journey (33.780 km) by foot, to retrace the pathways of our ancestors, the first humans who migrated from Africa and journeyed around the Earth - he posits that one must devote time to inter-personal connections in order to fully comprehend the human phenomenon *- “Out of Eden Walk*,” ^35^], we consciously stood for the concept of *“slow science”* for a slow pace of methodology. The small sample size does not allow to extrapolate far-reaching conclusions; however, it is noteworthy that the sample size reaches or exceeds that of previous basic psycho-neuro-immunological and is standard in basic fMRI studies. ^20^^-^^22^ Although patients were being treated for various illnesses while immersed in a clinical environment, the exact goal of the study was to know whether patterns marked enough to overwrite the heterogenicity of diagnoses could be elicited by the meetings. The diversity of the patients’ psychological and somatic conditions was managed by the encounter-centered study design, and the time interval between both data collections was relatively small (two hours), as compared to the length of the patient’s stay in the hospital (ranging from several days, up to weeks). The difference between the control and the intervention groups did not affect the main, significant result found with fMRI examinations, since that part of the study was performed within an auto-control setting.

### Psycho-immunological viewpoint

Although the measured immune changes were too small to be clearly distinguishable from other clinical effects, it is noteworthy that the results from the hospital bed show a striking similarity with those of the “ShoRT study”, where the children from whom blood samples were drawn in a non-painful way through branules, were visited by Smiling Hospital artists [SHoRT ^16^]. It was once again the more successful visits that generated more marked changes in lymphocyte counts. However, the growing inconsistency in lymphocyte changes as patients got closer to the chapel, and especially the results of the group receiving communion, could refer to the sense of fascination and admiration mixed with tones of fear, as reflected by the psychological or religious term *tremendum* (see Rudolf Otto’s terminology of the “numinous” and “mysterium tremendum et fascinans” to describe the experience of the holy ^36^). While the primary purpose of the intervention was to elicit positive emotions, the authors found that the same biblical reading could cause a wide range of thoughts and feelings, and different facets of the same emotional pattern could be amplified; this included fear of God, a recurrent motive of biblical encounters between God and man. For instance, in the Book of Exodus, God says to Moses: *“I will make all my goodness pass before thee … Thou canst not see my face: for there shall no man see me, and live.*” (Exodus 33, 19-20, KJV). More measurements are needed to clarify the statistical significance of these observations.

### Neurological viewpoint

First and foremost, it is worth noting that the most easily implementable technically, entailing the least possibility of error in the laboratory measurement, and automated lymphocyte count showed significant correlation with the fMRI results. This part of the study was not affected by the any unintended bias in the randomization process, since the fMRI part of the study was performed within an auto-control setting, for visited patients only. Yet, as Lieberman and Cunningham pointed out, whenever fMRI is used as a principal tool in a research setting, the number of measurements performed during each examination are so high that from a statistical point of view, even the use of the most conservative p values cannot rule out the possibility of type I errors, rendering the results of every fMRI study only meaningful following comparison with previous, and verification by subsequent studies, in case these also support the interpretations proposed. ^37^ The current paper discusses a plausible interpretation of the findings while further research is needed to confirm the results.

Given that we could not rule out potential effects of magnetic resonance on immunological parameters from the fMRI examination, we intentionally separated fMRI measurements from immunological measurements. We laid out the “block-design” setting we used based on a meta-analysis of 48 studies, ^38^ and works by Leff *et al.,* ^39^ and Beaucousin *et al.,* ^40^ who described different activation patterns on the fMRI of patients exposed to readings featuring different emotional content.

The fact that the comparison of the a-c and s-c periods resulted in a significant change in fMRI activity, whereas no difference was detected when comparing a and s, proves that the measurement was physiologically trustworthy: the patients heard the biblical readings, and control text likewise.

The biblical passage is an ancient, more than two and a half thousand years’ old text with plain words, and simple language structure, and is thought to have originally been intended to offer comfort. In contrast, the control text contained many difficult words for the layman, had long and difficult to follow sentences, and contained several words with potentially fear-inducing connotations. Thus, the result of the correlation analysis suggests the change in lymphocyte counts is related to the subjectively perceived content of the biblical reading as opposed to control text by the patients. The fact that the changes in lymphocyte counts were not significant, despite some widening in the confidence intervals, signaled that these changes were subject to limitations by the circumstances and by the disease; however, the correlation of these changes with the fMRI-s indicated that even when biblical readings did not appear to have a physiological effect, they could have an ordering, arranging effect, along some specific guiding principle, on a key parameter like the lymphocyte counts.

According to neurosynth.org, previous scientific publications related to the region in the 2 mm area of the -52, -56, 26 cluster, have linked this area to the tactile and manual reconstruction of shape recognition, the learning of words, emotional speech, and the encoding of belief systems in neural pathways, and their linking with ethical decision making. ^41^^-^^47^ It must be highlighted that the only brain area that showed a correlation with any of the immune parameters measured in this study - the left BA39, gyrus angularis - was contralaterally the same that is activated during meditation on/recitation of Buddhist scriptures. ^20^ Although the depth of understanding could only mean cognitive understanding, due to the core nature of the phenomenon observed and the study design, it can also refer to broader spiritual experiences – which, in turn, can only partially be influenced consciously. Thus, the brain area involved in the correlation described above (gyrus angularis) could also be linked to the *“*Aha! moment *”*, which contains cognitive and emotional components alike but is not, or only partially, subject to conscious influence.

### Excursus: theological perspectives

The authors would like to warn against interpreting the results of the study solely in terms of healing being accelerated by intimate religious experience. The spiritual, religious nature of the phenomenon observed obliges the integration of the theological viewpoint in the careful interpretation of the results. For centuries in the European tradition, theology – originally in close association with other areas of the basic scholar fileds " *septem artes liberales*" (seven liberal arts) - has been the delegated branch of academic studies as far as any aspect of the Bible was involved. More recently, modern research methodology has been embraced, especially by the reformed theologians of 20-21 ^th^ centuries’ academic Christian theology, rendering it a branch of science only differing from other specialties by the principal focus of its observations being the “Word of God” instead of the natural world. ^24^ This legitimizes the examination of how findings relate to the relevant theological literature in the discourse of a study involving religion, especially in the case of a study which used a biblical passage at its core.

When one is examining the effect of the Word of God on man, trying to correlate it with any human parameter, dialectical theologians immediately emphasize the inherent asymmetry in the relationship between God and man. As Tillich submitted, we cannot control God with our will: “The experience of Spiritual presence does something that the human soul in itself cannot” ^48^ Thus, the theologian points out that the immunological and neurological changes described in the measurement did not happened to the patient’s efforts, but without their knowing it. The content itself of the biblical message does not hold the promise of immediate physical healing. According to the Bible, Jesus’ healings give the impression that those had a signal value. Jesus did not heal everybody (cf. the account of the healing at Lake Bethesda, Jn 5, 1-9), and in a critical moment he himself accepted his own death. There are instances when Jesus’ Gospel has been theorized to give strength not just to overcome the disease, but to endure it. Nevertheless, correlation also exists in theology ***.*** Tillich wrote: “The answers emerging from the event of revelation are comprehensible only if they are in correlation with the questions of our whole existence, the existential question.” ^48^ Tillich used the method of correlation mainly in respect of the dialogue between the Christian message and the contemporary society (culture). The present paper describes how the method of correlation can also be used in the dialogue between natural sciences, social sciences and theology. Indeed, in Christian religion, the encounter between God and man can take place within a human relation, or also during a biblical reading, i.e., listening to the “Word of God”. ^24^ In this context, the meeting with the chaplain could trigger the recollection of some genuine, primordial experience of meeting with the transcendent, a phenomenon that could be the expression of ancient, maybe even pre-Christian patterns buried in our collective subconscious, and bringing up ancient, genuine, instinctive reactions, according to Jung’s concept on the working mechanism of “archetypes”, i.e., of inherited inner patterns buried in the collective subconscious. ^49^ The simultaneous change of the nervous and immune systems that was recorded in the present study during repeatedly listening to the Bible, confirms the possibility that in addition of the psychological effects, general biological patterns could also be activated during the meeting of God and man. In line with this concept, beyond the personal healing stories, the Bible’s synoptic contain numerous accounts of mass healings – i.e., Mt 4,24.8,16.12,15.14,14.15,30.19,2.21,14; Mk 6,56; Lk 4,40.9,11 – giving the impression that the meeting with Jesus also brought about general healing effects in people.

The contradiction presented above – namely, that the Word of God would be healing, whilst the main message of the Word sometimes does not bear healing, but cross – is solved if we put the question and the results of our study in the context of modern theologians’ evolutionary theory, which states that religiousness and life of faith also have developmental aspects, therefore can be understood as having an evolutionary dimension, with a direction of growth, at times including the notion of suffering through the process (see Whitehead ^50^ and “process theology”), and thus cannot be considered as opposed to the natural sciences’ concept of evolution.

According to Pannenberg, “at the end of the 19 ^th^ century in the first half of the 20 ^th^, sadly, Christian churches and theologians could not recognize that the teaching of evolution gives an unprecedented possibility for theology in regards of the possibility of its relationship with modern science. The fight against Darwinism was one of the mistakes resulting in the most serious consequences during the history of the relation of theology with sciences.” ^51^ The findings of the current study can be a small contribution to support this position, and put a novel lighting on the fact that, despite the lack of significant and noticeable biological effects, some form of religious belief could so indefatigably spread over and survive in the whole human species up until that day. If indeed, depending on the depth of spiritual understanding, the religious experience could influence the lymphocyte count, then it could contribute to prevent or recover from the diseases, hence provide an evolutionary advantage in the sense the natural sciences use this term. On the other hand, from the theologian’s perspective, the goal of evolution is not the survival and accommodation in the narrow sense of the word. Teilhard de Chardin wrote: “as early as in St. Paul and St. John we read that to create, to fulfill and to purify the world is, for God, to unify it by uniting it organically with himself”, and God is “from this point of vantage in the heart of matter, assuming the control and leadership of what we now call evolution.” ^52^ He states that “through human socialization, whose specific effect is to involute upon itself the whole bundle of reflexive scales and fibers of the earth, it is the very axis of the cosmic vortex of interiorization which is pursuing its course.” ^52^ Pannenberg often alluded to Gods’ appearance in world history, in the person of Jesus, as an event that can be considered the portent, the anticipation of the future. ^53^ In this sense, healings are of signal value because they hint a precursory picture of a harmonious God-man relationship, including all its psychical and physical aspects – as Pannenberg wrote: if, “(like Teilhard de Chardin), we can consider life’s evolution as the process of the creation of life forms of increasing complexity and at the same time becoming increasingly introspective, then we can also state, that in the succession of different forms of life by the creatures, is expressed the increase in the shareholding of the divine Spirit, of life’s Spirit.” ^51^^,^^52^

Faith is an evolutionary advantage, in the theological sense because the goal of evolution is the increase in the shareholding of the divine Spirit. ^52^ This is the end towards which the result of our measurement converges.

## Data availability

### Underlying data

Figshare: Healing Study Beres et al. - Part 1: Psychological and Immunological Changes after meeting a Chaplain coupled with Biblical Readings among Hospitalized Patients, https://doi.org/10.6084/m9.figshare.16750384.v1 ^54^

Figshare: Healing Study Beres et al. - Part 2: fMRI Changes after meeting a Chaplain coupled with Biblical Readings and Recalling the Visits with Audiotapes among Hospitalized Patients, https://doi.org/10.6084/m9.figshare.16751851.v1 ^55^

### Extended data

Figshare: HEALING Study Beres et al. - F1000Research - extended data, https://doi.org/10.6084/m9.figshare.17029715.v1 ^56^

### Reporting guidelines

Figshare: CONSORT checklist for “Healing through faith: meeting a chaplain coupled with biblical readings could produce lymphocyte changes that correlate with brain activity (HEALING study)”, https://doi.org/10.6084/m9.figshare.17029715.v1 ^56^

Data are available under the terms of the “figshare” repository (licence CC BY 4.0).

Data generated from the above raw data are included in this article.
