# Healing through faith: Meeting a chaplain coupled with biblical readings could produce lymphocyte changes that correlate with brain activity (HEALING study)

**DOI:** 10.12688/f1000research.74504.5

**Published:** 2024-10-30

**Authors:** András Béres, Miklós Emri, Csaba Aranyi, Dániel Fajtai, Ferenc Nagy, Péter Szabó, Pál Bödecs, Edit Hörcsik, Éva Perpékné Papp, Ferenc Tomanek, Márta Kuti, Ágnes Petőfalviné, Hajnalka Kisdeákné, Gergely Bíró, Dániel Kovács, Bettina Bakos, Eszter Vinczen, Eszter Gál, Renáta Sillinger, Zoltán Szalai, Antal Szilágyi, Marianna Kiss-Merki, György Nagyéri, Judit Fodor, Tamás Németh, Erzsébet Papp, Imre Repa

**Affiliations:** 1 Pápa Reformed Theological Seminary, Pápa, Hungary; 2 Kaposi Mor Hospital, Kaposvár, SOMOGY County, Hungary; 3 University of Debrecen, Debrecen, Hungary; 4 Dr. József Baka, Diagnostic, Radiation Oncology, Research and Teaching Center, Kaposvár, SOMOGY County, Hungary; 5 Reformed Church in Hungary, Kiskunhalas, Barcs, Kaposvár, Hungary; 6 Hungarian Catholic Church, Kaposvár, Hungary; 7 SoftFlow Hungary Ltd., Pécs, Hungary

**Keywords:** faith, hospitalization, psychoneuroimmunology, theology, fMRI

## Abstract

**Background:**

Faith and belief systems impact the emotional as well as immunological states of believers in ways that we are just beginning to understand. However, the clinical implications of prior studies are limited.

The aim of the HEALING (Hospital-based Ecumenical and Linguistic Immuno-NeuroloGic) study is to examine immunological and neurological changes in hospitalized patients after meeting with a chaplain coupled with the study of biblical readings.

**Methods:**

Hospitalized patients were pre-screened to identify those who were most in need of a spiritual intervention. A passage from the Bible was read to them during a meeting with the chaplain at bedside (n = 20) or in the chapel (n = 18). No meeting occurred in the randomized control group (n = 19). Blood samples were obtained 30 min prior and 60 min after the meeting to measure white blood cell (WBC) count, interferon-gamma (IFN-γ), immunoglobulin M (IgM), IgA, IgG, and complement 3 (C3). A subgroup of the visited patients was subjected to functional magnetic resonance imaging (fMRI), during which they listened to an audiotape of readings of the same biblical passage (n = 21).

**Results:**

Immunological changes were not significant. Conversely, a significant (p _fwe_ = 0.003) correlation was observed between lymphocyte changes and activation of the angular gyrus (left BA39) during fMRI, a brain area involved in word recognition.

**Conclusions:**

This article contributes to the relevant literature by helping to create a realistic picture of the possibilities of neuroimmune modulation in clinical practice. Compared to healthy volunteers, the extent of short-term neuroimmunomodulation becomes narrower in a clinical setting. Although limited by the sample size and cohort study design, the findings suggest that the depth of psycho-immunological changes could depend on the degree to which the chaplain’s main message is understood.

**Abbreviations**
BA: Brodmann area
fMRI: functional magnetic resonance imaging
NK cells: natural killer cells
NSAID: nonsteroidal anti-inflammatory drug
For other abbreviations, see Table 1.
Hungarian translation - Magyar fordítás: See *Extended data.*

## Introduction

Following Selye’s description of how stress modulates the immune system, ^1^ numerous studies have shown a direct and complex relationship between acute, ^2^^–^^5^ chronic stress and the immune system, ^6^^–^^9^ with evidence revealing the long-term effects of early life stress on the immune response. ^10^^–^^12^ Far less research has examined the modulating effect of positive emotions on immunity. Berk *et al.* reported signs of immune stimulation in healthy adults after watching humorous videos. They displayed a 60-min humorous video to two groups, each consisting of 10 healthy adult men. They observed an increase in immunoglobulin levels (IgM, IgA, and IgG), activated T-cells (the total number of T cells as well as the Th-, Tc- and naive T cells separately), the proportion of lymphocyte subgroups, and peripheral IFNγ level. In six men, they observed that when NK-cell activity increased, it was statistically significant (IgM: *p* < 0.09; IgA: *p* < 0.01; IgG: *p* < 0.02; T-cell number: *p* < 0.01; IFN-γ-level: p = 0.02; NK-cell activity: *p* < 0.01). Most of the changes peaked 30 min and 90 min after the intervention began and were still measurable the next day, 12 h afterward. ^13^ Takahashi *et al.* showed a 75-min humorous video to 21 healthy adult volunteers and noted a significant ( *p* < 0.05) increase in NK-cell activity. ^14^ Following these early findings, we posited that the short-term effects of positive emotions would primarily be manifested in immune stimulation (as seen in an increased number or activity of immune parameters), as opposed to the negative effect of chronic stress, which causes immune suppression. As further research has evolved, a more nuanced picture has developed.

In 2001, Bittman *et al.* held approximately 60-min group therapy sessions involving percussion music for 30 adults and reported a significant increase in NK cell activity ( *p* = 0.055). However, such an increase did not occur after every session—only after composite drumming, and there was no measurable change in IL-2 and IFN levels. This was one of the first studies to draw attention to the importance of patient selection and the quality of interventions. ^15^

Later, Bennett *et al.* suggested that the extent of immunological changes related to interventions intended to elicit positive emotions may largely depend on how the emotions are subjectively perceived. After showing a humorous video to 16 healthy women, they did not find significant changes in NK-cell activity compared to the control group, except when they included the level of cheerfulness in their calculations ( *p* = 0.037). They measured this by counting the number of pre-established metacommunicative signals (from simply smiling to laughing out loud), which indicated the amount of mirthful laughter elicited among the participants (the Humor Response Scale). ^16^

Delving further into the phenomenon of positive emotions affecting the immune system, we broadened the scope of the study to include sick people, that is, participants whose immune systems were not intact when the study began. This is especially challenging because certain conditions are associated with immunosuppression while others are linked to chronic inflammation, underlining the importance of context when assessing which immunological changes can be considered positive. In 2001, Burns *et al.* conducted a music therapy session for 29 adults at a cancer help center using both recorded and live music and observed an increase in secretory IgA levels in both cases. ^17^ In 2005, Matsuzaki *et al.* showed a 60-min live session of *Rakugo* (a traditional Japanese tale) to 41 adults with rheumatoid arthritis and observed significant changes in their IL-6, TNF-α, IL-4, IL1Ra-citokine, and cytokine receptor antagonist levels ( *p* < 0.05) within 10 min of finishing the story. Changes in pro- and anti-inflammatory markers differed and could be related to the severity of the disease. ^18^

In 2007, Hayashi *et al.* showed a 60-min humorous video to six men and four women with type 2 diabetes and observed a significant change in postprandial glucose levels as well as an increased expression of many genes, including those that regulate NK cell activity. ^19^ Similar studies have traced how positive experiences can trigger immunological changes in a chain reaction down to the genetic code, creating “molecular signatures” related to mind–body interventions. ^20^

Most of the basic studies cited above were conducted with healthy adults in non-clinical conditions using non-personal tools (humorous videos) in the context of group sessions; hence, their clinical relevance is limited. As such, we aimed to evaluate the potential immunological effects of personal interventions intended to elicit positive emotions at the bedside of hospitalized patients. In the daily routines of hospital wards, this involves artists visiting sick children and chaplains visiting adults. Previously, the SHoRT (Smiling Hospital Research Team) study examined the immunological impact of positive emotions spurred by the positive experiences of sick children treated in a hospital. ^21^ In the HEALING (Hospital-based Ecumenical and Linguistic Immuno-NeuroloGic) study described below, we tried to elicit a positive emotional effect by meeting with a chaplain in a pre-selected adult population. With the current study being representative of a new direction in clinical psycho-neuro-immunological (PNI) research, ^22^^,^^23^ we decided to record the psychological and immunological changes related to the intervention as well as employ functional magnetic resonance imaging (fMRI) to detect neurological events.

Intended to elicit positive emotions to alleviate the burden of being hospitalized and facilitate healing among patients, chaplains perform a spiritual function by representing the religious institutions from which they originate. Hence, the effects of their work on patients contain rich religious undertones.

Research on religion and spirituality as therapeutic factors is a scientific field in its own right. There is now a general consensus that these experiences are beneficial for health, including physical aspects. ^24^ At the same time, pioneering researchers in the field early drew attention to the fact that due to the nature of the spiritual phenomena, they cannot be researched in exactly the same way as in other fields, “as simply another garden-variety topic for sophisticated analysis.” ^25^ In addition to characteristics that depend on cultural context, ^26^ the historical period, crises in particular, ^27^ and to a certain extent age (interestingly emphasizing the role of a time window in adolescence, ^28^ midlife, ^29^ and old age ^30^), the mere orientation of the spiritual experience towards transcendence raises more fundamental, epistemological questions concerning the limits of how far scientific knowledge can or should progress in this direction at all. ^31^

Research on how religious experiences impact health—especially physical health—is a developing field. Prior studies have only explored the long-term impacts of religious life on the immune system ^32^^–^^34^ or relevant brain areas, ^35^^–^^38^ while neurological events have been examined in isolation from other physical changes. Moreover, the concept of religious practice occurring in a hospital setting is a sensitive issue, posing many practical difficulties (e.g., “God at the bedside”). ^39^

The above studies with healthy volunteers indicate (especially with regard to the modest statistical p values measured, even among healthy volunteers) that producing changes in immune parameters through spiritual means is not an easy task. Taking into account financial and logistical limitations as well, we aimed to determine whether—with arrangements similar to prior studies using the above sample sizes—we could measure relevant immunological changes that significantly influence patient recovery in a clinical setting. With the HEALING study, we aimed to examine whether general PNI patterns could emerge from a single spiritual encounter within a clinical environment or whether changes measured in previous studies were lost in the sea of other factors affecting the immune system.

Based on the abovementioned literature, one could expect to detect at least subtle immunological changes following a visit by a chaplain and activation of brain areas such as the medial frontal gyrus, lateral middle frontal gyrus, angular gyrus, and supramarginal gyrus (similar to the areas activated by meditation) ^35^ in the current study, which involved patients listening to and subsequently recognizing a sacred text (i.e., a biblical reading). With regard to the correlations linking immune parameters with fMRI recordings, it is reasonable to expect a large number of type I errors given the study design, which was prepared to relate the relatively limited database of immune parameters to the robust database of all brain activities recorded. We attempted to address this challenge by comparing our findings with those of previous studies.

Our trial is registered at www.ClinicalTrials.gov (Identifier: NCT04112121, Registration date: October 2, 2019).

## Methods

### Study design

We used a randomized, parallel, open-labeled, controlled clinical trial design:

1. The effect of biblical readings on immunological parameters
  ○ **HEALING I. Measuring the effect of biblical readings at the bedside:** The core measurement occurred at the first meeting with the chaplain, coupled with biblical readings by the patient’s bed. We tried to evaluate if the spiritual intervention of “acceptance of the Word” elicited immunological as well as psychological changes, as evaluated by laboratory measurements and questionnaires. (In Christianity, listening to a passage from the Bible is considered a way of encountering God. Thus, in Christian terminology, the phrase “Word of God” refers either to a specific biblical passage, to the Bible in general, or directly to God. ^40^ Certain terms or phrases used in this article that refer to faith/the Bible will be placed in quotation marks.) The first blood sample was collected 30 min before the visit; the second blood sample was drawn 120 min after the first sample. We evaluated 20 patients.
  ○ **HEALING II. Measuring the effect of biblical readings at the hospital chapel:** For this segment, the biblical readings took place in the hospital chapel in small groups. We included 18 patients. The same biblical passage used in the previous setting was used for reading.
2. The effect of biblical readings on fMRI activity

We recruited patients from the previous two measurements for this portion of the study based on their mobility and fMRI availability. During this portion, patients listened to biblical readings again (the same passages they first heard during one of the past two measurements). Passages alternated between a control text and a period of silence. We focused on whether any of the immunological or psychological parameters that appeared to change after the first listening session correlated with changes in fMRI activity.

**Figure 1.**
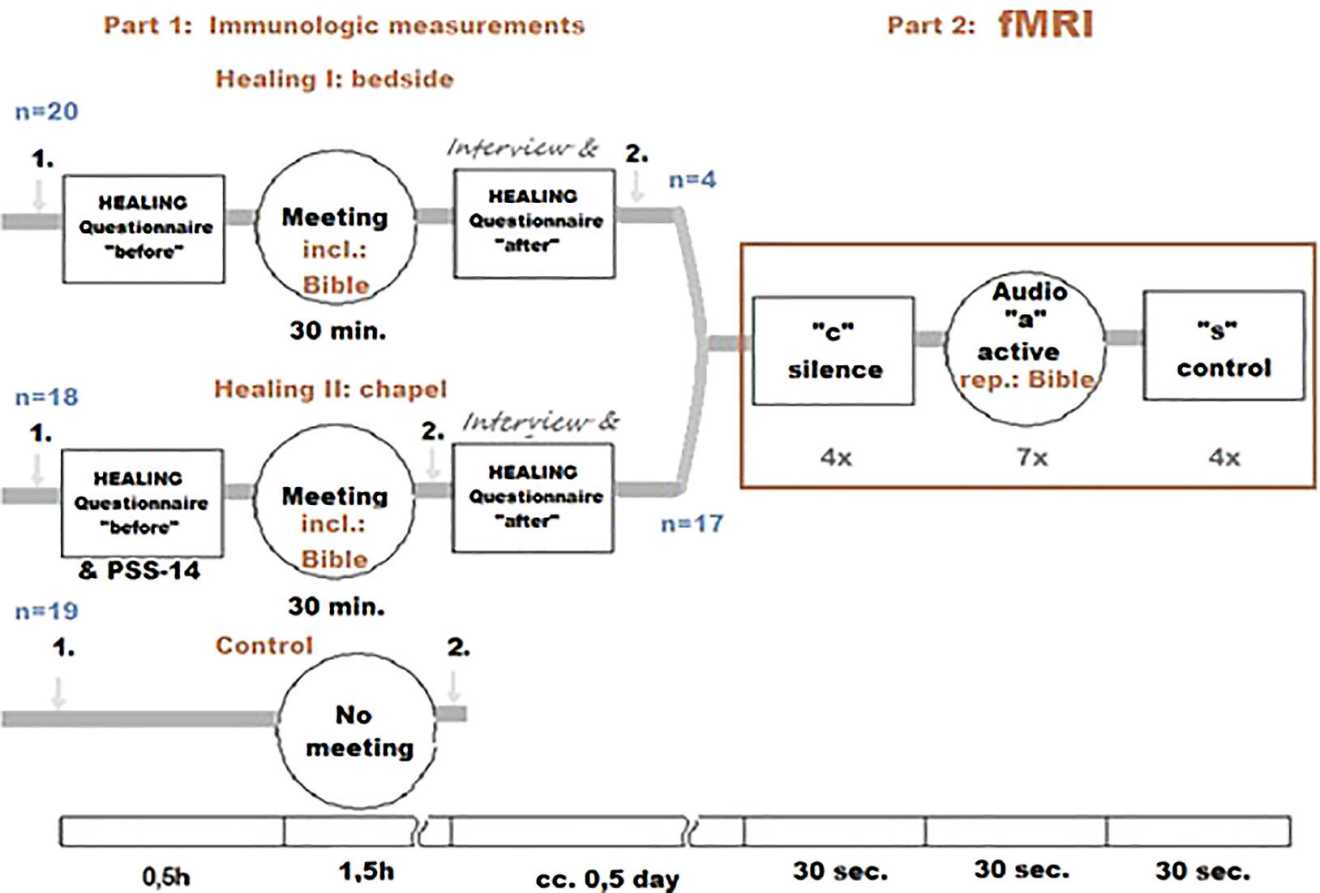
HEALING study design. 1. and 2.: blood samples.

For a graphic overview of the study design, see Figure 1.

We screened a total of 351 patients for eligibility and randomized 60. We excluded data from three patients because their medical condition required acute use of oral non-steroidal anti-inflammatory drugs (NSAIDs)/metamizole sodium. We analyzed results for a total of 20 patients in the bedside group (with an enrollment rate of up to two patients per week, i.e., HEALING I), 18 patients in the chapel group (with an enrollment rate of three to five patients per week, i.e., HEALING II), and 19 patients in the control group. We analyzed data from 57 patients in total. Of these, 22 underwent fMRI. We reported a technical failure in analyzing fMRI data from one patient (the first patient in the chapel group), although the event did not recur. We observed no other exclusions or loss of data after randomization.

We obtained measurements in the chapel (n = 18) in five independent groups (each group: minimum = 3 patients; maximum = 5 patients). In three of the five groups (11 patients), we gave the patients the option to receive communion: the Catholic Eucharist or the Reformed Lord’s Supper. Five of the 11 patients opted to receive the Catholic Eucharist (four patients) or the Reformed Lord’s Supper (one patient).

Patients were recruited from September 10, 2015, to January 2, 2017; the nature of the interventions did not require follow-up after January 2, 2017. No changes were observed in the trial outcomes or methods after the study commenced. The study was terminated when the number of planned enrollments was reached. We identified no harmful or unintended effects in the patients during or after the study.

For an overview of enrollment, see the CONSORT Flow Diagram of the HEALING study (cf. Figure 1 in the *Extended data*).

The protocol was approved by the Hungarian Medical Research Council’s Committee for Research Ethics (approval #7245-1/2014/EKU [55./2014], authorization #SOR/074/00130-4/2014) and the internal ethical boards of our institutions (IG/02013-003/2015; 270/2015). We obtained written informed consent from all participants and conducted the study in accordance with the principles of the Declaration of Helsinki.

### Enrollment

The inclusion criteria were adult age (>18 years), hospitalization, ability to communicate verbally, alertness, orientation, no signs of psychosis in their medical history, and willingness to participate in the study after giving written informed consent. The hospital’s infectious disease and nephrology wards were involved in the recruitment process. We proposed enrolling all patients satisfying the above criteria. Mobility was an inclusion criterion for events in the chapel and fMRI measurements. Due to the limited availability of chaplains, not all eligible patients were able to participate. The decision was based on the patient’s degree of need and willingness as assessed by and at the discretion of the chaplain using a quick stratification scoring system designed to address the practical needs of this study (the HEAL Score, see Appendix 1, *Extended data*). The HEAL Score (which consists of the sum of four scales ranging from 1 to 4 each) is based on the hospital chaplain’s/hospital staff’s intuition of the neediest patients (“H4”), the patients who self-reported the highest need for visits (“E4”), the least religious patients (“A4”), and the patients with the most serious illness (“L4”). Exclusion criteria were the inability to communicate verbally, psychotic state (as reported by the physician responsible for the patient), altered mental state, unwillingness to participate, active and treated malignant disease, steroids, use of NSAIDs, or use of metamizole sodium, since these could have influenced the measured immunological parameters.

Random assignment was based on the availability of a chaplain on the day of the measurement rather than chance allocation of all patients willing to be visited; thus, the results of the control group were not biased by disappointments or frustrations caused by the cancellation/postponement of an anticipated visit. As such, the atmosphere in the control group reflected the genuine psychological environment of a common day at the hospital undisturbed by ordinary events. The randomized control group consisted of patients who knew the goal of the measurement but were explicitly asked to help with their participation in the control group; that is, they knew they were controls and that they were not going to meet the chaplain (upon request, the encounter could be scheduled for a later occasion).

The investigator arranged for enrollment. To minimize allocation bias, covariate-adaptive, blocked, stratified randomization was used: Block size was fixed to 19 (±1) enrolled patients for each group, with a 1:1 allocation ratio. For the control group, we enrolled patients whose diagnoses and number of days in the hospital were similar to those of the intervention groups to ensure a good balance of participant characteristics since the intervention groups were saturated.

### Intervention and measurements

***Personal encounters with the chaplain, listening to a biblical passage, and psycho-immunological measurements***

**Figure 2.**
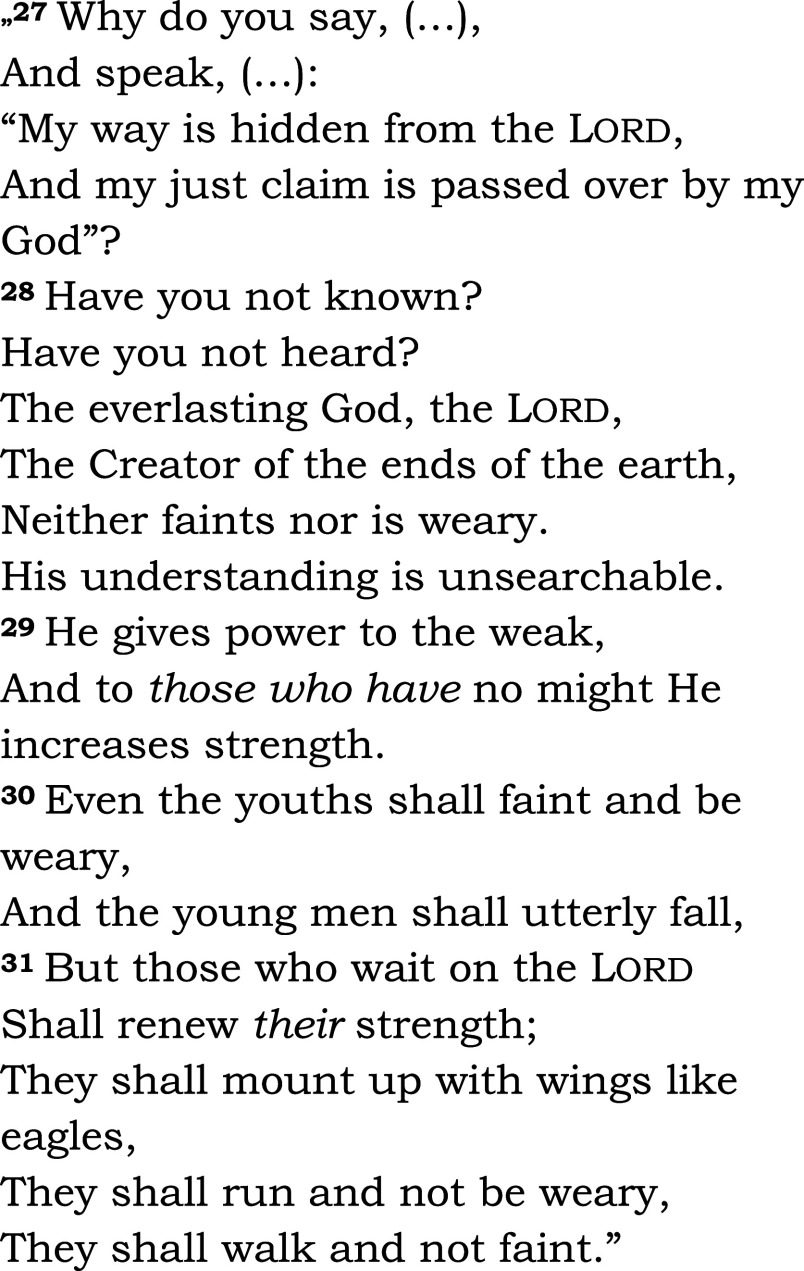
Read aloud to all patients in the bedside or chapel groups, and during subsequent fMRI-s. Passage listened to by patients at bedside individually or in groups at the chapel as well as on audiotape during the fMRIs. Isaiah 40:27-31. New King James Version (NKJV). In this study, patients heard readings from the chaplain in their native language, Hungarian.

Both groups listened to the same passage (Isaiah 40, 27–31, Figure 2). In the group where patients were visited at bedside, the chaplain provided personalized, supportive spiritual therapy in which, after listening to the patient’s current situation and reflecting on it, she tried to integrate a predetermined, encouraging verse into the conversation. In the group that met in the hospital chapel, the chaplain delivered the same short sermon to all participants at the same time, which was based on the same biblical verse. Among the five groups in the chapel, communion was offered to three: the Eucharist for Catholics and the Lord’s Supper for members of the Reformed Church. We asked the patients and chaplains to complete a questionnaire designed for the study (the HEALING questionnaire, preliminary pilot testing; see Appendix 2, *Extended data*). For the groups in the chapel, we used the validated score of the Perceived Stress Scale, which has 14 items (PSS-14). ^41^ We collected blood samples 30 min before and 60 min after the encounter due to some anticipated differences in the length of the visits, which lasted about 30 min each. The time interval between the two samples was fixed at 120 min. We supplemented the lab measurements with microscopic examination of the blood smears ^42^^–^^44^ ( Figures 4, 5, and 6), measurements of IFNγ- (HEALING I) or immunoglobulin M-, A-, G-, and C3-levels (enzyme-linked immunosorbent assays), and a few blood clotting factors (partial thromboplastin time [PTT] and international normalized ratio [INR]) (HEALING II).

**Figure 3.**
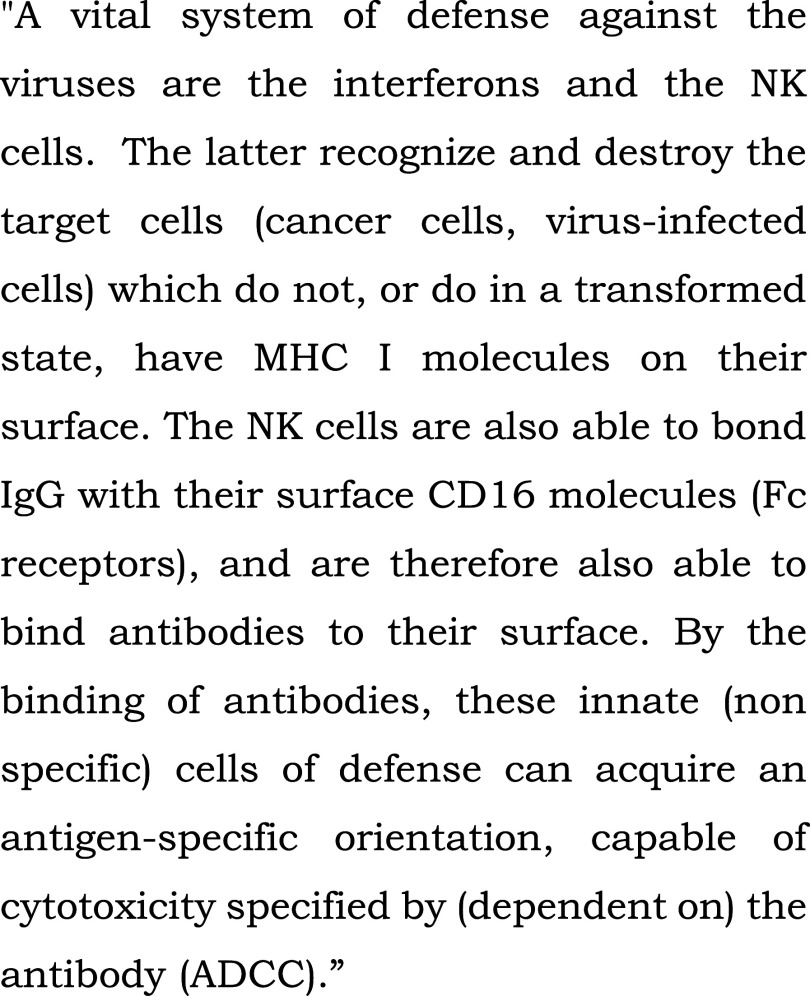
Control text read to all participants during the fMRIs in the HEALING Study. Control text listened to on audiotape by the patients (either coming from the bedside or the control groups) who underwent fMRIs. “Innate (non-specific) immune system” (extract). From: Szalka A, Timár L. Infektológia [Infectology]. Budapest: Medicina; 2005. Patients heard the text read by the chaplain in their native language, Hungarian, in the study.

**Figure 4.**
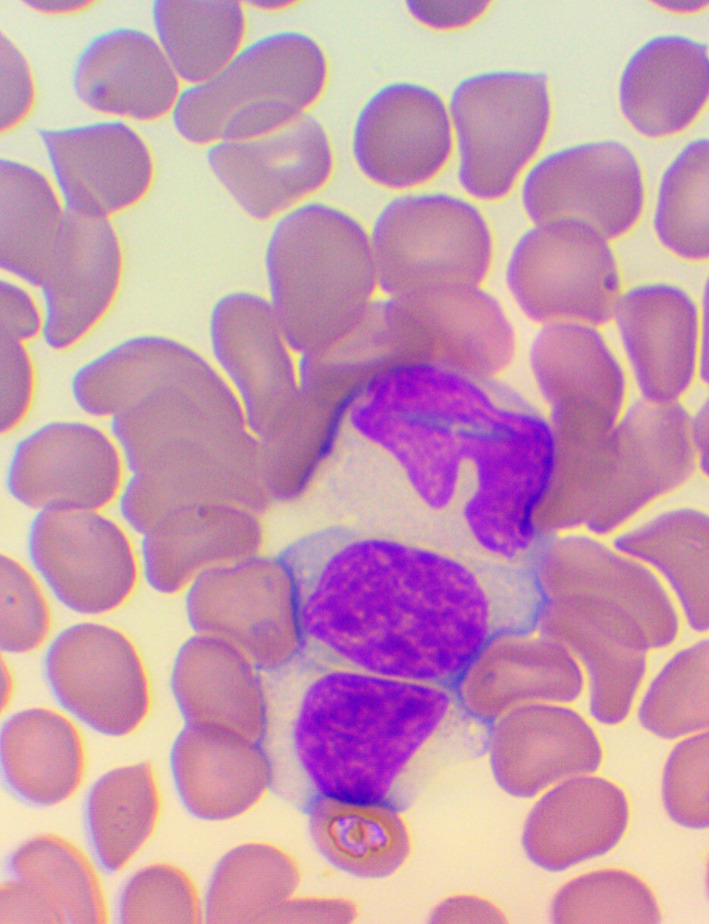
Activated lymphocytes: Microscopic examination of blood smear in the HEALING study.

**Figure 5.**
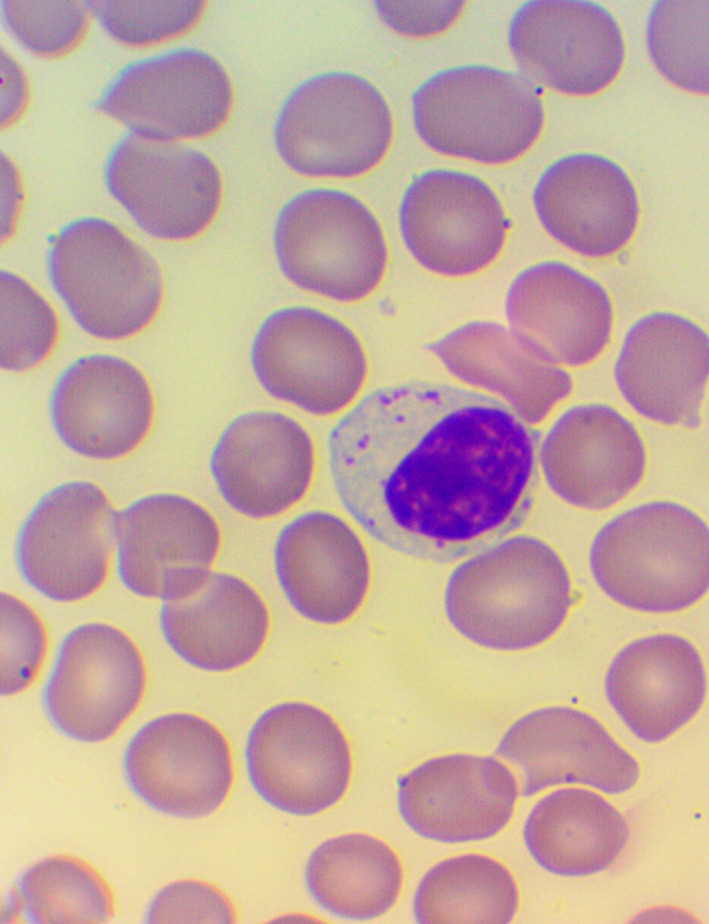
Large granular lymphocyte (LGL): Microscopic examination of blood smear in the HEALING study.

**Figure 6.**
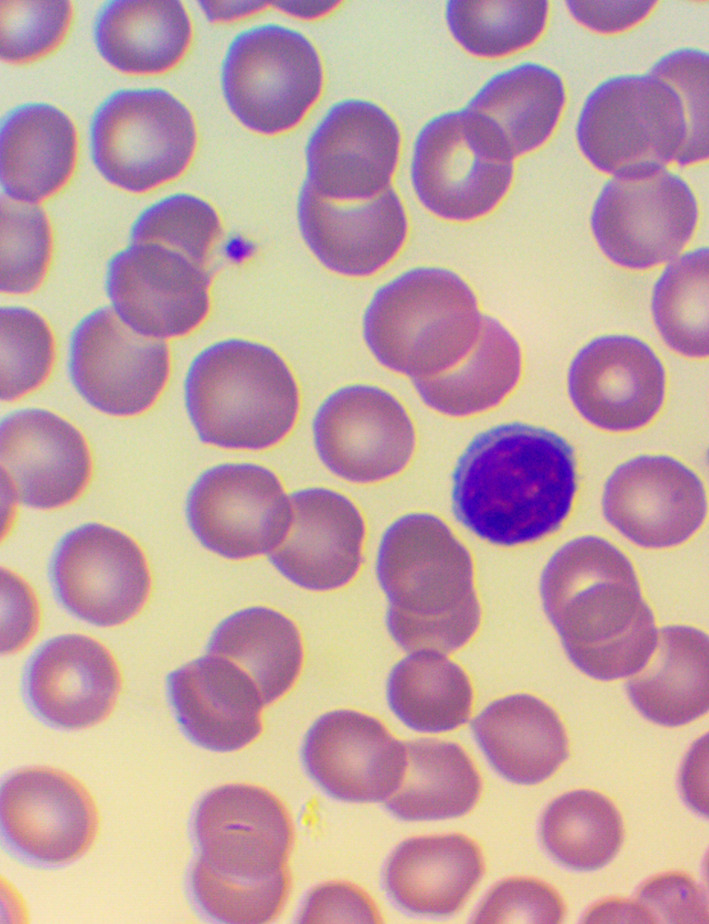
Small condensed lymphocyte: Microscopic examination of blood smear in the HEALING study.

For an overview of the measured psychological factors, immunological parameters, and corresponding abbreviations, see Table 1.

**Table 1.** Psychological factors and immune-hematological parameters assessed in the HEALING study.

Psychological factors	Immune-hematological parameters
Based on the patient’s answers to the HEALING “before” questionnaire: • Patient’s personal belief on how she/he got sick (BI1 and BI2) • Patient’s desire for the visit (BII1, BII4) • Patient’s satisfaction with her/his life (BI3, BI4, BI5, BI6) • Patient’s self-assessed burden caused by hospitalization (BI7, BIV) • Patient’s personal belief on the way she/he will be healing (BII2, BII3) • Patient’s personal faith (BII1) • Patient’s religious practice (BIII2) Perceived stress scale 14 items (PSS-14) Based on the patient’s or the chaplain’s answers to the HEALING “after” questionnaire: • Patient’s/Chaplain’s overall satisfaction with the visit (AI and PI, respectively) • Patient’s assessment on the intimacy of the encounter (AII) • Chaplain’s assessment on the patient’s openness (PII) • Patient’s assessment on the sincerity of the encounter (AIII) • Chaplain’s assessment on the extent she could connect to the patient (PIII) • Patient’s assessment on the trustworthiness of the chaplain (AIV) • Patient’s/Chaplain’s assessment on the emotionally turbulent, roiling effect of the encounter (AV, PIV) • Patient’s/Chaplain’s assessment on the emotional depth of the encounter (AVI, PV) Abbreviations refer to the question number in the corresponding HEALING questionnaire	• White blood cell (WBC) count • Neutrophils Changes in: ✓ neutrophil count (dNeut) ✓ percentage of neutrophils (dNeut%) • Eosinophils Changes in: ✓ eosinophil count (dEo) ✓ percentage of eosinophils (dEo%) • Basophils Changes in: ✓ basophil count (dBas) ✓ percentage of basophils (dBas%) • Monocytes Changes in: ✓ monocyte count (dMono) ✓ percentage of monocytes (dMono%) • Lymphocytes Changes in: ✓ lymphocyte count (dLy-abs) ✓ percentage of lymphocytes (dLy%) ✓ percentage of large granulated lymphocytes (dLGL%) ✓ percentage of small condensed lymphocytes (dSmallLy%) ✓ percentage of activated lymphocytes (dMiddleLy%) • Lymphocyte/neutrophil ratio Change in: (dLy/Neut) • Platelet count (Plt) • Interferon-gamma level (IFNγ) • Immunoglobulin M level (IgM) • Immunoglobulin A level (IgA) • Immunoglobulin G level (IgG) • Complement C3 level (C3) • Partial thromboplastin time (PTT) • International normalized ratio (INR)

For nominal values, we used non-parametric, associative tests (i.e., Kolmogorov–Smirnov). Regarding immunological parameters, we used a normality test followed by a parametric, paired-samples t-test. For all parameters measured in the study, we performed a network analysis employing two different methods: a Bayesian analyzer developed at the University of Technology and Economics (BME) in the Faculty of Electrical Engineering and Informatics in Budapest, ^45^ and the R package IsingFit (R code used: IsingFit [data, family = “binomial,” AND = TRUE, gamma = 0.1, plot = TRUE, progressbar = TRUE, lowerbound.lambda = NA, vsize = 10]), ^46^ followed by correlation analysis. We divided the commonly used *p*-value (0.05) by the number of comparisons analyzed (Bonferroni correction).

***Biblical passages read repeatedly on audiotape and fMRI measurements***

Subsequently, we performed fMRI examinations, which were contingent upon the availability of fMRI and the capability of the patients to be mobilized. After providing written informed consent, the patients receiving fMRI were comforted to prevent any possible anxiety related to the measuring environment (narrowness and loudness often pose a challenge for patients). They were given instructions on the process and then laid down on the fMRI equipment, where they could hear the passage read aloud on an audiotape. They were allowed to stop the examination at any time by pressing a button.

To identify the regions of the brain that the current measurement could impact, we conducted the fMRI examinations using a block-design technique in three functional states:

1. In the active biblical passage (“a”) block, patients could listen to the same passage they had heard in their hospital bed or in the hospital chapel. They heard the passage read by the chaplain once again in their native language (Hungarian) with a modern translation ( Figure 2).
2. In the scientific control (“s”) block, the stimulus was a scientific text from an audiotape, also read by the chaplain ( Figure 3). Although this text was intelligible, it contained many difficult scientific words and complex grammatical structures in Hungarian (a Finno-Ugric language), which posed an intellectual challenge for patients.
3. For a reference state, we introduced a block of patients exposed to silence (“c”).

The fMRI examinations were performed using a 1.5T Siemens Magnetom Avanto MR scanner (Syngo software versionVB17/A, Siemens Medical Solutions, Erlangen, Germany); for the timing of the stimulation and synchronization of data collection, we used the software Nordic Aktiva v1.1. (Nordic Neurolab, Bergen, Norway). For all enrolled patients, we obtained a structural 3D T1-weighted axial MP-RAGE recording (TE = 4.73 ms, TR = 1540 ms, TI = 800 ms, flip angle = 15°, slice-thickness 0.8 mm, 0.9 × 0.9 × 0.9 mm voxel-size) with a 3-s repetition time. We obtained a blood oxygenation level-dependent (BOLD) recording sequence (T2* gradient echo, TR = 3000 ms, TE = 42 ms, flip angle = 90°, interleaved 4 mm axial slice thickness, 3.6 × 3.6-pixel size) with 145 components. During the fMRI measurements, the stimulation always started with a 15-s block of silence, followed by seven sections of activation blocks for 60 s each. The latter constituted a 30-s active and a 30-s control section or silence. During the measurements, we performed the “block design” type stimulation in a “c → as → ac → as → ac → as → ac → as” sequence order for all patients.

In the first phase of processing the fMRI image database, we assigned the T1-weighted structural image transformation into the MNI152 atlas space using the FSL 5.0 and ANTS 1.9 programs. ^47^^,^^48^ Using the segmentation algorithm of the FreeSurfer 5.0 software package ^49^ in native space, we created the brain-T1 pictures, which only contained the images originating from the surface of the brain. Using the brain-T1 pictures, we transformed the motion-corrected fMRI picture sequences into the T1-picture corresponding to the person in question and then transformed it into the MNI152 atlas space. ^47^^,^^48^ Finally, for all fMRI sequences, after eliminating the first four recordings containing the T1-effect, we applied 8-mm isotropic Gaussian filtering. We used SPM12 software ^50^ to perform statistical analyses for the created fMRI picture database at the individual and population levels. While processing the individual fMRI image sequences using the a-c, a-s, and s-c contrasts, we generated statistical image databases (contrasting pictures) showing the differences between the effects of the various stimuli, which we employed in the population-level analysis to statistically characterize the effect of the stimuli. Finally, we examined the differences in BOLD-answers linked to the active and control audio stimuli and their correlation with concrete clinical data corresponding to each patient. In the SPM analysis, to compare the statistical differences between the a-c, c-a, a-s, s-a, c-s, and s-c activities, we sorted the activation clusters containing a minimum of 100 voxels, with a Student-t = 3.58 threshold corresponding to the non-corrected *p* < 0.001 value from the SPM {T} pictures. To characterize them, we then used the MNI152 spatial coordinates of the cluster maximum, the maximal t-value, the corresponding family-wise error rate (FWER)-corrected probability (peak-level inference), the size of the cluster, and the FWE-corrected probability of cluster occurrence (cluster-level inference). ^50^

## Results

The enrollment period lasted for one year. In total, we analyzed 57 patients. Their median age was 64 years (HEALING I), 65 years (HEALING II), and 66 years (control). The only criterion when enrolling patients for the control group was that the patient’s age, type of disease, and days of treatment did not show considerable differences compared with the intervention groups. Due to the small number of patients willing to participate, we needed to use statistical stratification with constraints (cf. Table 1, *Extended data*).

### Part 1: Psycho-immunological changes measured after the meeting with the chaplain

As for whether they believed in God, most patients (HEALING I: 65%, HEALING II: 77.8%) answered positively. Only 25–38.9% of the patients said they actively practice their faith. A total of 35% and 44.4% of the patients reported cathartic or a very positive experience at the end of the measurement (a maximal rating of 5/5 to Question “AI” in the HEALING “after” questionnaire, i.e., the patient’s overall satisfaction with the visit, as reported on a single-item rating scale); 60% and 66.7% reported the visits to be deeply emotional (a minimum rating of 4/5 to Question “AVI” in the HEALING “after” questionnaire, i.e., the patient’s assessment of the emotional depth of the encounter, as reported on a single-item rating scale).

Changes in immunological parameters were not statistically significant after the Bonferroni correction. While we checked all possible correlations between the psychological and immunological parameters, no significant correlation emerged (see the visualization of all correlations in Figure 5, emphasizing the stronger psycho-immunological correlations in Figure 6; *Extended data*).

### Part 2: Results of the fMRI measurement

We obtained evaluable fMRIs from a total of 21 patients in the second part of the study.

The comparison of the “active biblical reading (a)” block with the “reference silence (c)” block showed significant ( *p* < 0.001) activation in the right and left BA 22 (Wernicke’s) and the right and left BA41 (primary auditory) areas only *.* The comparison of the “scientific control (s)” block with the “reference silence (c)” block revealed significant ( *p* < 0.001) activation in the same areas only. The comparison of the “active biblical reading (a)” block with the “scientific control (s)” block during fMRI did not show any demarcated brain area that would have significantly different activity among all patients (Figures 2–4, *Extended data*).

**Figure 7.**
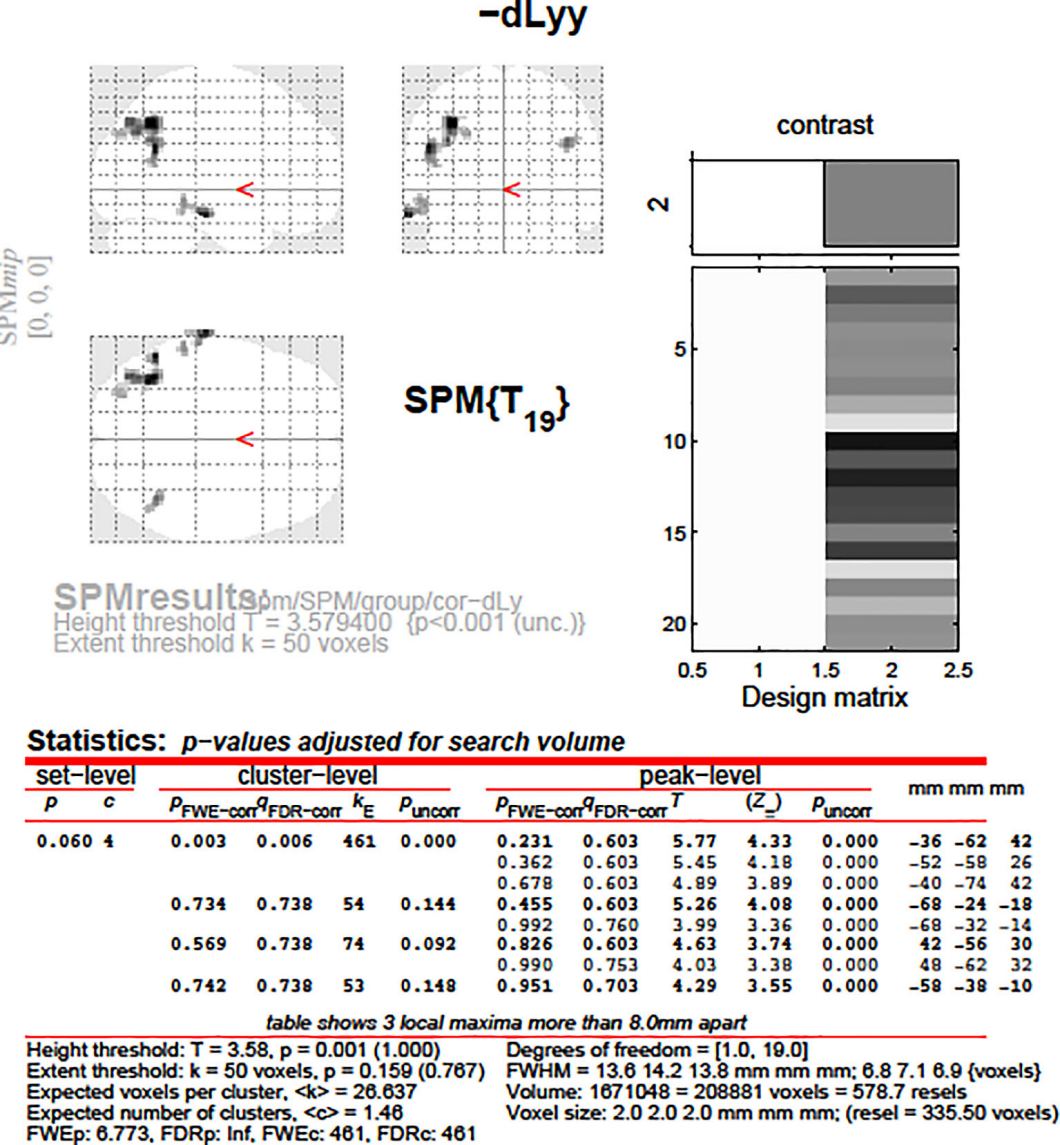
dLy-abs vs. a-s fMRI in the left BA39. Change in lymphocyte count (“dLy”) versus the difference between activation during the active biblical reading “a” and the scientific control “s” block (“response”) during fMRI in the left BA39 *.*

**Figure 8.**
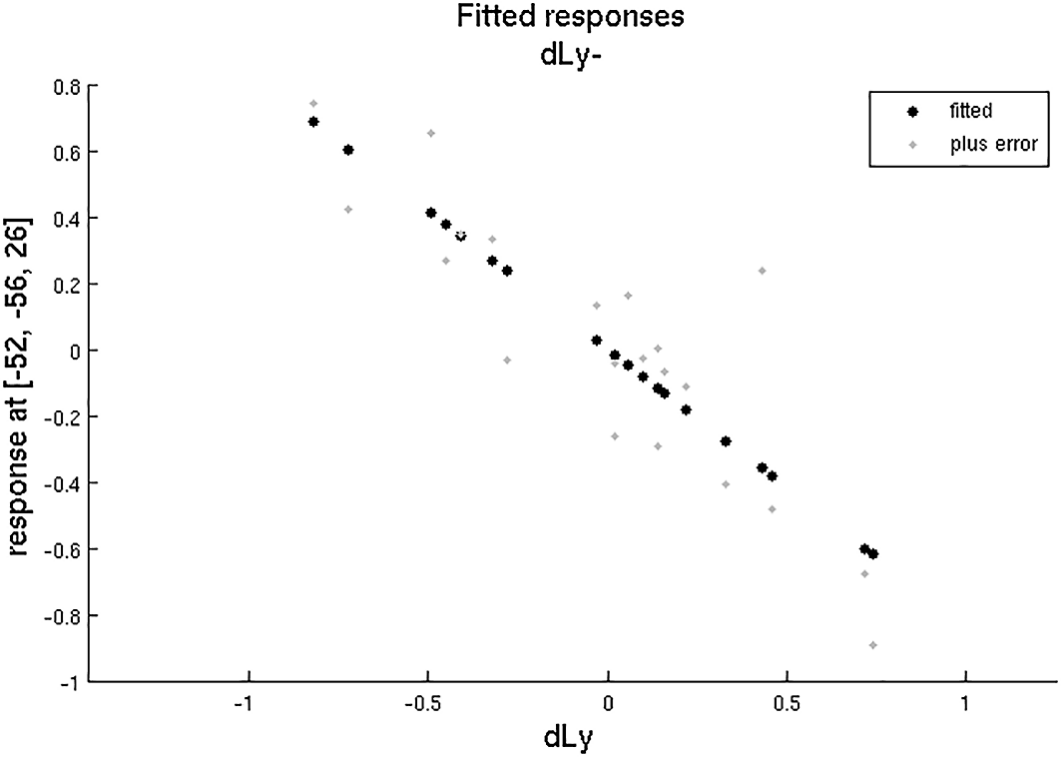
dLy vs. fMRI in the left BA39. Change in lymphocyte count (“dLy”) versus the difference between activation during the active biblical reading “a” and the scientific control “s” block (“response”) during fMRI in the left BA39 ( *p* = 0.003, r = -0.9584).

After that, we performed several subgroup analyses to explore the correlations between the psychological factors or the immunological parameters and the changes in fMRI activity between the “active biblical reading (a)” and “scientific control (s)” blocks. We categorized the subgroups of patients according to the extent of the change that we measured in the psychological factors—i.e., the chaplain’s overall satisfaction with the visit [“PI”] and the patient’s overall satisfaction with the visit and assessment of the emotional depth of the encounter [“AI,” “AVI”], as reported on the single-item rating scales corresponding to the HEALING “after” questionnaire—or the immunological parameters. The latter entails changes in the lymphocyte count (dLy-abs), the percentage of lymphocytes (dLy%), and the lymphocyte/neutrophil ratio (dLy/Neut) (as measured in the laboratory), along with changes in the percentage of LGLs (dLGL%) (as measured with microscopic examination of blood smears). These subgroups contained approximately similar (half and half) numbers of patients.

Among the dLy-abs subgroups, there was a tendency toward a change in fMRI activity. We observed a weak difference in activity in the area of the left BA39 between the subgroup of patients who showed an increase in lymphocyte count and those who exhibited no relevant increase in lymphocyte count ( *p* = 0.393). No other psychological or immunological pairs of subgroups displayed any differences with regard to changes in fMRI activity.

After that, we aimed to correlate the dLy parameters with the areas showing changes in fMRI activity (still for the changes in fMRI activity between the “active biblical reading (a)” and the “scientific control (s)” blocks only). In Figure 7, the darker brain areas indicate a stronger correlation of brain activity with the number of lymphocytes. An inverse linear correlation emerged ( *p* = 0.019 with dLy%, *p* = 0.003 with dLy-abs) between the change in activity of the left BA39 and the change in lymphocyte count ( Figures 7 and 8; *p* = 0.003, r = -0.9584). Finally, we performed the correlation analysis for the change in fMRI activity between the “active biblical reading (a)” and “scientific control (s)” periods and the dNeut, dLy/Neut, AI, AVI, and PI factors or parameters, but we observed no significant correlation.

Accounting for correlation and subgroup analyses, we examined only 12 factors or parameters; hence, the significance level was 0.05/12 = 0.00417. The correlation between the left BA39 area and change in lymphocyte count remained significant after Bonferroni correction.

## Discussion

### Neurological viewpoint

The most easily implementable technique—entailing the least possibility of error in the laboratory measurements—the automated lymphocyte count showed a significant correlation with the fMRI outcomes. Unintended bias did not affect this part of the study’s randomization process because the fMRIs were performed in an auto-control setting for the visited patients only. However, as Lieberman and Cunningham pointed out, whenever fMRI is used as a principal tool in a research setting, the number of measurements obtained during each examination is excessive from a statistical perspective. Even using the most conservative p-values cannot rule out the possibility of type I errors. This makes the outcomes of every fMRI study meaningful only after comparing it with past studies and verifying it with subsequent research. ^51^ We discuss a plausible interpretation of the results, but further investigation is needed to confirm them.

Since we could not rule out the potential effects of magnetic resonance on immunological parameters from the fMRI examination, we intentionally separated the fMRI and immunological measurements. We laid out the “block-design” setting we used based on a meta-analysis of 48 studies, ^52^ including Leff *et al.* ^53^ and Beaucousin *et al.,* ^54^ who described different activation patterns in the fMRIs of patients exposed to passages read aloud that featured different emotional content.

The comparison of the “a”-“c” and “s”-“c” periods revealed a significant change in fMRI activity in the primary auditory areas, whereas we detected no such difference when comparing “a” and “s.” This proves that the measurement was physiologically trustworthy: the patients *heard* the biblical passages and the control text.

Since we intentionally left ample opportunity for spontaneity and subjectivity, it is not surprising in hindsight that no distinct brain area emerged when comparing the “a” and “s” periods. As stated above, spontaneous meetings with a person of faith are the most subjective, the most diverse, and hence the least generalizable part of the spectrum of spiritual experiences. We would have observed general differences in brain areas between participants hearing a religious recitation and a neutral/scientific recitation if the intervention group had been composed of participants highly trained in meditation (such as Buddhist monks). The participants of the HEALING study were ordinary people from various religious backgrounds, which probably resulted in more subjective differences in the way they perceived the sacred texts read aloud to them.

We could not unanimously detect any general differences among all enrolled patients between the biblical and control blocks; therefore, hearing a religious recitation did not affect the brain differently than a neutral/scientific recitation. However, a significant correlation emerged between lymphocyte count and brain activity when subtracting the scientific control block from the biblical block. This outcome echoes previous psycho-immunological results mentioned in the introduction, especially those of Bennett *et al*., ^16^ who detected no general immunological changes following the presentation of a humorous video to healthy volunteers but observed a significant correlation with changes in immune parameters when they found a way to quantify how the patients subjectively viewed the video. They employed a humor response rating scale that corresponds to the fMRI results in our study, thus representing different tools used for the same purpose: to measure individual differences in how interventions are perceived.

Contrary to our expectations, although the database of immune parameters was many times scarcer than the enormous database of whole brain activity, remarkably, we did not obtain obvious false positive results. When comparing the “a” and “s” periods, the lack of generally distinguishable areas of activation reduced the chance of deriving type I errors related to dense activation patterns. Only a single correlation emerged, suggesting that it was reasonable not to automatically consider this result a spurious effect. The biblical passages constituted the sole fixed, generalizable element of the visits; consistent with this, the only brain area that correlated with any of the measured immune parameters was involved in understanding these passages.

The biblical passage used is an ancient (more than two-and-a-half thousand years old) text with plain words and simple syntax; it is thought to have originally been intended to offer comfort. In contrast, the control text contained many words that would be challenging for the layman to easily grasp; moreover, it had long sentences that were difficult to follow and several words with potential fear-inducing connotations. Thus, the results of the correlation analysis imply that the change in lymphocyte count is related to the patients’ subjectively perceived content of the biblical passage as opposed to the control text. The fact that the changes in lymphocyte count were not significant (despite some widening in the confidence intervals) indicates that these changes were subject to limitations by the circumstances and disease. However, the correlation of these changes with the fMRIs suggests that even when biblical passages do not seem to have a physiological effect, they could have an ordering or arranging effect along a specific guiding principle based on key parameters such as lymphocyte count. In other words, although the effect size of the lymphocyte changes was too small to produce significant changes under the limited sample size of this study, the amplitude of the changes appeared to vary with the evolution of a single parameter: activation of the left angular gyrus.

According to neurosynth.org, previous scientific publications related to the region in the 2-mm area of the -52, -56, and 26 clusters have linked this area to the tactile and manual reconstruction of shape recognition, the learning of words, emotional speech, and the encoding of belief systems in neural pathways and their connection with ethical decision-making. ^55^^–^^61^ The only brain area that showed a correlation with any of the immune parameters measured in this study—the left BA39 (gyrus angularis)—was contralaterally the same as that activated during meditation or recitation of Buddhist scripture. ^35^ Although the depth of comprehension could only denote cognitive understanding, due to the core nature of the phenomenon observed and the study design, it could refer to broader spiritual experiences, which in turn contain cognitive and emotional components and are not (or only partially) subject to conscious influence.

### Psycho-immunological viewpoint

Contrary to our expectations, grounded in a preliminary scientific review of the literature relating faith to psycho-neuro-immunology, ^62^ no significant psycho-immunological results emerged. We assume one reason for this is that the study could have been underpowered regarding its sample size, given the small size of the immunological changes observed. Another reason could be that most patients—although truly grateful for being visited—were not motivated to meet with a chaplain to have a transformative experience. If we turn our attention from mass healing to the personal accounts of healing found in the New Testament—such as those of a man with leprosy (Mt 8, 2–4), a sick woman who was bleeding (Lk 8, 43–48), and the blind (Mk 10, 46–52)—the healed seem to share at least a few of the following traits:

• Their conditions were *critical,* often compounded by remorse and loneliness.
• The sick themselves or a close relative *strongly wanted* them to be healed.
• Jesus seemed like the ultimate hope, a last chance, as seen in the following:
  ○ “ *What do you want me to do for you?”* (Mk 10,51).
  ○ *“Your faith saved you”* (Mk 5,34).
  ○ *“If* I *can do something? Everything is possible for the one who believes”* (Mk 9,23); see *Grün et Robben.* ^63^

The people healed in the biblical accounts describe their encounters with Jesus as *transformative.* However, statistically speaking, the psychological effect was of much lower importance for the patients enrolled in the present study. Despite the thorough enrollment process, only 40% of the patients in the bedside group and 5.5% of patients in the chapel group were given an “H4” Score (cf. HEAL Score above). This indicates that even among the “chosen” in the pre-screened group of patients whom we evaluated with the HEAL Score, the chaplain was certain that only a tiny fraction of patients needed to be visited. The PSS-14 scores revealed that the patients’ average level of stress in the month preceding measurement was not significantly high (the PSS-14 had a mean value of 27/56), and their responses to the Healing Questionnaire showed that they did not perceive the psychological or physical wounds incurred in their daily lives to be deep (see tables 2, 3, and 4, *Extended data*).

As for whether the patients felt responsible for their own chronic ailments, blaming oneself was the only psychological factor that displayed a correlation (albeit a weak one) with any immunological parameter (see the answer “BI2_b,” i.e., “It is my fault that I became sick; I blame myself …”). This was one of the rarest answers among the patients (10% to 22% of the patients in the bedside [HEALING I] and chapel [HEALING II] groups, respectively). In other words, by the time the chaplain arrived for a visit, most patients were not experiencing personal difficulties and did not view the visit as a life-altering event. Patients were open to the visit but were mostly concerned with feeling exhausted and needing help during their hospitalization.

**Figure 9.**
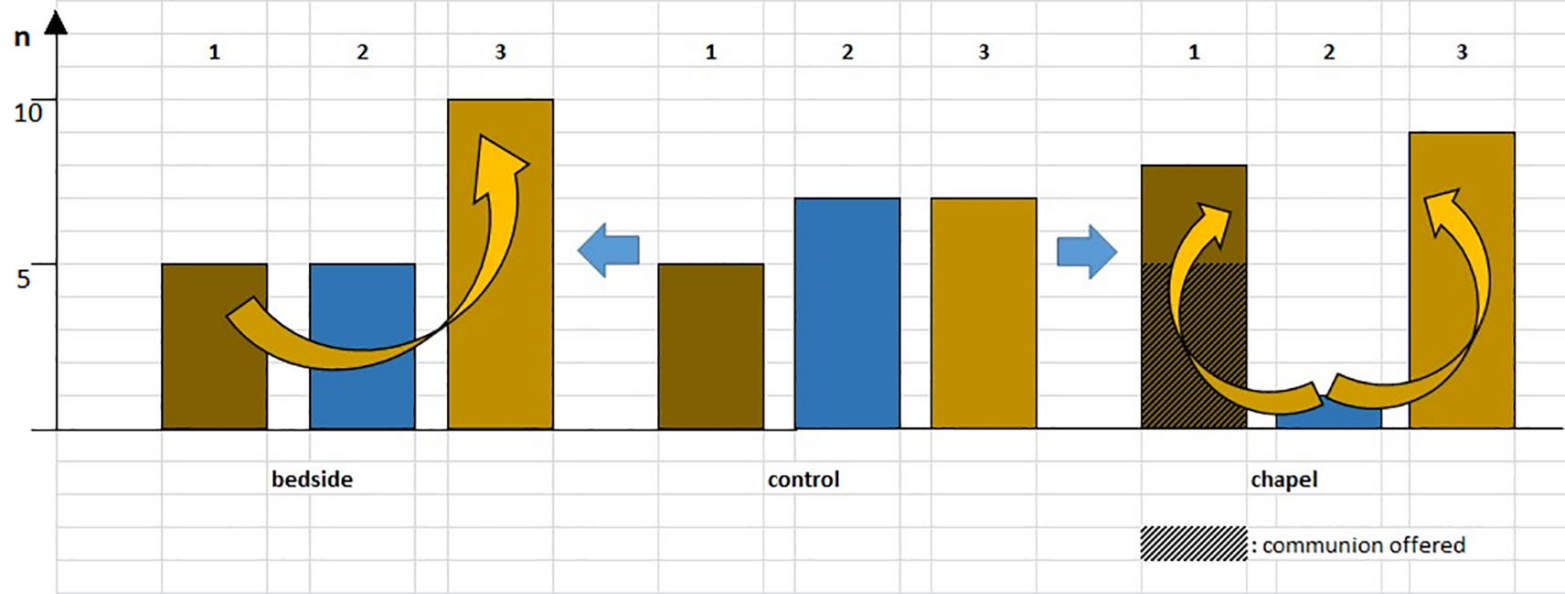
Changes in lymphocyte count in the HEALING I (bedside) and HEALING II (chapel) studies. The columns indicate the number of patients. 1 = dark brown: lymphocyte count (Ly-abs) and percentage (Ly %) decreased; 2 = blue: Ly-abs and Ly % did not change in the same direction; 3 = light brown: Ly-abs and Ly % increased. Taking the control group as the baseline condition (center), the blue arrows suggest two possibilities for eliciting change: intervention at the bedside (the arrow to the left) or intervention in the chapel (the arrow to the right). Yellow arrows denote possible trends.

**Figure 10.**
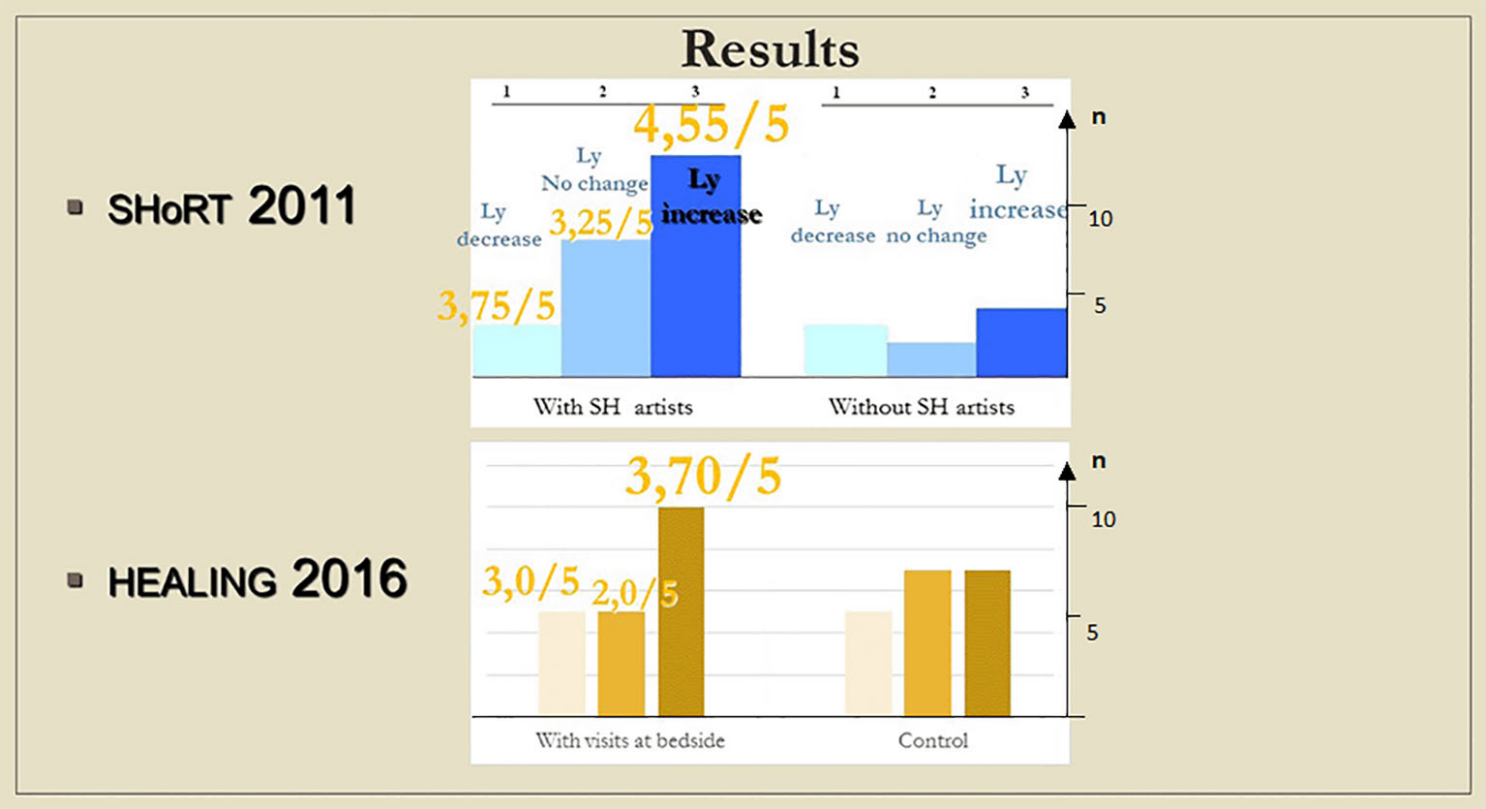
Number of patients with a lymphocyte count decreased, with no change, and increased. In case a meeting took place, the numbers above the columns indicate the average of subjective scores (from 1 to 5) by which the artists (SHoRT) or the chaplain (HEALING) evaluated the encounter.

In order to verify finer deviations, future research needs to clarify that although these changes were not statistically significant after the Bonferroni correction, some trend-like deviations (consistent with prior literature) appeared, suggesting the study may have been underpowered in terms of sample size. Indeed, changes in lymphocyte count appeared to exhibit trend-like deviations in both groups (at bedside and in the chapel; Figures 9 and 10). Also, for future research, the personal, one-on-one meetings (not the group sessions) consistently produced an insignificant but considerable small effect size in lymphocyte changes across the measurements. The effect size for dLy-abs was d = 0.23 at the bedside (HEALING I, individual) but d = 0.09 in the chapel (HEALING II, group). The effect size for dLy-% was d = 0.28, p = 0.08 in terms of laboratory testing, and d = 0.31 with p = 0.053 for microscopic examination of blood smears at bedside (see the p-values of immune system changes in tables 2, 3, 4, *Extended data*).

In Figure 10, the columns are grouped in threes; in each group, from left to right, the height of the column shows the number of children with a decrease, no change, and an increase in lymphocyte count, respectively. At the top of the columns, the patients’ visitors’ subjective assessment of the visits is shown on a 5-point scale (higher scores denote more successful visits; at the time of the assessment, the visitors did not know the results of the laboratory measurements). As the figure demonstrates, although the measured immune changes were too minimal to be distinguishable from other clinical effects, the results from the bedside encounters revealed a striking similarity with those of the ShoRT study, in which Smiling Hospital artists visited children from whom blood samples were drawn in a non-painful way through branules (SHoRT). ^21^ Once again, more successful visits generated more marked changes in lymphocyte count.

Figure 9 draws attention to another interesting observation. The columns indicate the number of patients, with the color of the columns referring to the direction of the change in lymphocyte count (1 = dark brown: the lymphocyte count (Ly-abs) and percentage (Ly %) decreased; 2 = blue: Ly-abs and Ly % did not change in the same direction; 3 = light brown: Ly-abs and Ly % increased). Taking the control group as the baseline condition (center), the blue arrows suggest two possibilities for eliciting change: intervention at bedside (the arrow to the left) or intervention in the chapel (the arrow to the right). The yellow arrows imply possible trends; it appears as if the closer the patients got (physically and psychologically) to the chapel—especially regarding the results of the group receiving communion (without exception in this subgroup, we recorded a decline in lymphocyte count, which is considered a sign of stress in the PNI literature; see Figure 9)—the growing inconsistency in lymphocyte changes seemed to reveal a sense of fascination and admiration mixed with tones of fear, as reflected by the psychological/religious term *tremendum* (see Rudolf Otto’s terminology of the “numinous” and *mysterium tremendum et fascinans* to describe holy experiences). ^64^ While the primary purpose of the intervention was to elicit positive emotions, we observed that the same biblical passage could trigger a wide range of thoughts and feelings and that different facets of the same emotional pattern could be amplified, including fear of God, which is a recurrent motif for biblical encounters between God and humans. For instance, in the Book of Exodus, God says to Moses: *“I will make all my goodness pass before thee … Thou canst not see my face: for there shall no man see me and live.*” (Exodus 33, 19-20, KJV). More measurements are required to map the main emotional themes at play during meetings with a chaplain and clarify the statistical significance of these observations.

### Limitations

***Our study has several limitations.***

First, the small sample size does not allow for the extrapolation of far-reaching conclusions; however, the sample size reaches or exceeds that of previous PNI studies ^13^^–^^17^ and is standard in basic fMRI research. ^35^^–^^37^

Second, the patients were treated for various illnesses while immersed in a clinical environment. However, our goal was to determine whether meetings with a chaplain could elicit patterns marked enough to overwrite the heterogeneity of diagnoses. The diversity of the patients’ psychological and somatic conditions was managed by an encounter-centered study design, and the time interval between both data collections was relatively small (2 h, following the previously mentioned literature’s ranges for time intervals) ^13^^,^^16^^,^^18^ compared to the length of the patient’s stay in the hospital (ranging from several days to weeks). The difference between the control and intervention groups did not affect the main, significant outcome found in the fMRI examinations since we performed that part of the study within an auto-control setting; that is, only patients previously visited by the chaplain received an fMRI, and we did not compare their results to those of the non-visited group. Instead, the patients who underwent fMRI examinations while listening to the biblical passage read aloud were also exposed to the scientific text read aloud as well as their own reference states of silence that served as controls.

Finally, the limitations of all portions of the current study on spirituality are related to general concerns about their reproducibility. The study design of the measurements addressed this issue by placing strong emphasis on assuring that the encounters remained as authentic as possible for the intervention groups (e.g., leaving ample opportunity for the chaplain’s spontaneity) and by reproducing the genuine atmosphere of a usual day in the hospital for the control group (open randomization based on the availability of a chaplain instead of chance allocation, which could lead the patients to become disappointed).

The term “spiritual experience” refers to a vast range of collective and personal experiences, and its neural aspects (the “neural correlates of spirituality”) seem to depend heavily on the study design that is used to record them (cf. “Mystical experience in the lab”). ^65^ As Andersen *et al.* stated, “while studying powerful mystical experiences in believers in a controlled environment would enable researchers to identify, isolate, and analyze the central aspects of the phenomenon, we need an experimental setting with the power to elicit spontaneous mystical experiences.” In line with this, the spatial delimitability of the spiritual experiences’ neural imprint appears to depend on whether the nature of the experience itself is based on the more objective (e.g., monks reciting Buddhist scripture) ^35^ or more subjective (e.g., nuns recalling the most intense mystical experience they have ever had) ^66^ end of the spectrum. While an abundance of relevant literature elaborates on what happens with the “self” during *meditation*, ^67^ *mindfulness*, ^68^ or *prayer*, ^36^ we intentionally investigated a different area of spiritual experience by conceptualizing it in terms of meeting with a significant other. We assert that the spiritual experience is fundamentally not (or not merely) a different state of mind but the opportunity to transcend one’s “self” through meeting a significant other (in the case of this study, a chaplain). Although we cannot rule out additional secondary effects of meditation or prayer when a chaplain visits a patient (at times, patients are occasionally allowed to pray or meditate on a biblical passage), we did not focus much on the impacts of these adjunct elements but instead on the involuntary influences of an interactive, direct meeting with a person of faith.

In the spiritual care approach, the hospital chaplain facilitates the relationship with the transcendent Other. Hypothetically speaking, let us assume that, through scientific examination of the spiritual experience, it may be understood as both an intra-personal and inter-personal event between the individual and the significant, transcendent Other. Let us also assume that measurement tools of the natural sciences (since they are *immanent* to the natural world) might not be suitable for measuring the *transcendent* Other. As such, the scientific approach is limited in terms of providing an appropriate form of measurement and may detract from the authentic nature of spiritual encounters. When using the scientific approach by itself, we may be content with simply measuring (and even then, only to a limited extent) the impact of an inter-personal encounter on the individual, the intensity of one’s experience with the transcendent Other, the subjectively perceived presence of the transcendent Other, etc. without grasping or exercising control over the transcendent Other.

When examining the impact of a religious intervention on the self, we generally consider it necessary to ensure spontaneity (by allowing the chaplain’s intuition to prevail), authenticity (in the present measurement, using the original biblical passage), and personalization (with the chaplain’s personalized bedside meeting).

The main methodological challenge of this study is that genuine meetings with chaplains in hospitals always contain a spontaneous (“inspired”) element. Right from their initiation, these authentic meetings are always based on the chaplain’s intuition (which includes a range of elements, from which patient she visits to the advice she gives). This spontaneous aspect has a major impact on the entire meeting and, in turn, any PNI imprint (if there is any). The ample opportunity left for the chaplain’s inspiration reflects the original setting of biblical times in which the “Word of God” was first said when such meetings happened as a spontaneous act of the participants. This is how Jesus met people. Had we removed this spontaneous element, we would have prevented the occurrence of the phenomenon for which we were trying to estimate the potential PNI imprint. The question that arose was whether meetings with a chaplain could elicit immunological changes in patients *if* these meetings were allowed to be as authentic and spontaneous as they once were in biblical times. This methodological decision to leave the spontaneous aspect in did not affect the reproducibility of the results.

We suggest replicating the findings by proposing to schedule a meeting for *every* patient available in a ward on the day of taking measurements, by establishing a rough order for the visits (the “HEAL Score”, cf. above) to help manage the time available (but always letting the chaplain ultimately decide which patients to visit), and asking the chaplain to use the same biblical passage in every spontaneous, personalized meeting.

Journalist Paul Salopek trekked 21,000 miles (33.780 km) on foot to retrace the pathways of the first humans who migrated out of Africa and spread around the globe. He posited that one must devote time to cultivating interpersonal ties to comprehend the human phenomenon fully; he later created the non-profit “ Out of Eden Walk.” ^69^ Considering his “slow journalism” concept as a model, we advocate for “slow science” to ensure a gradual pace for methodology. Together with the ecumenical atmosphere of the study, the consciously slow pace of enrollment reinforced the authenticity of the encounters. For example, for two patients who were difficult to transport, we brought them to their actual beds by elevator to the hospital chapel, located on one floor beneath the Infectious Disease Ward. This experience evoked the biblical scene of the four men who brought their paralytic friend down from the roof, lowering him with cords, to help him reach Jesus through the crowd (Mk 2,4).

### Conclusions and clinical relevance

Our results contribute to the relevant literature by helping to paint a realistic picture of the extent of short-term immune changes that can be induced by spiritual means in the clinical environment. The results indicate that, compared to healthy volunteers, at least when it comes to the immune parameters measured in the daily routine, the possibilities of neuroimmune modulation in the clinical environment become narrower.

Our results point to a brain area whose activity changes along with lymphocyte count while meeting with a chaplain, coupled with listening to biblical passages in a hospital setting (for an excursus on theological perspectives, see below). Provided they accept that visits cannot be forced, nor that the outcome of the meetings is fully anticipated, physicians inviting a chaplain to meet with their patients should consider these visits as an additional factor that could exert an influence on the patients’ lymphocyte count and hence alter their recovery from the disease, depending on the depth at which the chaplain’s core message is understood.

### Excursus: Theological perspectives

We caution against interpreting our results solely in terms of healing that is accelerated by an intimate religious experience. Our measurements touch upon the religious sensibilities raised by the topic. We must elaborate on them from a theological angle to avoid potential distortions in interpreting our findings. The results raise deep questions: *Why* did we not measure more immunological changes if the “Word of God” is believed to heal people? If lymphocyte count is correlated with the part of the brain involved in understanding words, does that mean that those who *try* to understand biblical passages better will have more healing lymphocytes? These are theological questions. As such, the spiritual and religious nature of the phenomenon observed requires integrating the theological viewpoint for careful interpretation of the results.

For centuries in the European tradition, theology—originally closely associated with the *septem artes liberales* (the seven liberal arts)—has been the chosen branch of academic studies as far as the Bible is concerned. More recently, modern research methods have been embraced, especially by the reformed Christian theologians of the 20 ^th^ and 21 ^st^ centuries, rendering it a school of thought that only differs from the methodology of any branch of scientific cognition by focusing on the “Word of God” instead of the natural world. ^40^ This legitimizes examining how our findings relate to the relevant theological literature in the discourse of a study involving religion, especially in the case of using a biblical passage.

When exploring the effect of the Word of God on humans and trying to correlate it with human parameters, dialectical theologians immediately emphasize the inherent asymmetry in the relationship between God and people. As Tillich asserted, we cannot control God with our will: “The experience of spiritual presence does something that the human soul in itself cannot.” ^70^ In light of this, it is important to underline that we cannot claim that the immunological and neurological changes observed in our measurements were due to each patient’s own efforts; rather, such changes are traces of experiences that happened to the patients. Another important theological statement is that the content of the biblical message as a whole does not hold promise for immediate physical healing. According to the Bible, Jesus’ healings occurred rarely, which implies that they had a signal value. Jesus did not heal anything himself (cf. the account of the healing at Lake Bethesda, Jn 5, 1-9), and at a critical moment, he accepted his own death. There are instances where Jesus’ Gospel has been theorized to give strength to overcome disease and to endure it. Nevertheless, a correlation between God and healing exists in theology *.* Tillich wrote: “The answers emerging from the event of revelation are comprehensible only if they are in correlation with the questions of our whole existence, the existential question.” ^70^ Tillich used correlation mainly with respect to the dialogue between Christian messages and contemporary society (i.e., culture). This study describes how correlation can be employed in dialogue between the natural sciences, the social sciences, and theology. In Christianity, encounters between God and humans occur within a human context, including while listening to a biblical passage (i.e., listening to the “Word of God”). ^40^ In this way, a meeting with a chaplain could trigger the recollection of a genuine, primordial experience of meeting with the transcendent. This phenomenon could express ancient (maybe even pre-Christian) patterns buried in our collective subconscious, bringing up ancient, genuine, instinctive reactions according to Jung’s concept of the working mechanism of “archetypes,” i.e., of inherited inner patterns buried in the collective subconscious. ^71^ The simultaneous changes in the nervous and immune systems that we recorded during repeated instances of listening to the Bible suggest that, in addition to psychological effects, general biological patterns could also be activated during meetings between God and humans. In line with this concept, beyond personal stories of healing, the Bible’s Synoptic contains numerous accounts of mass healing (i.e., Mt 4, 24.8, 16.12, 15.14, 14.15, 30.19, 2.21, 14; Mk 6, 56; Lk 4, 40.9, 11), giving the impression that meeting with Jesus produced general healing effects in people.

We can solve the above contradiction—i.e., that the Word of God may be healing while its main message sometimes does not lead to healing—if we place the question and our results in the context of modern theologians’ evolutionary theory. It states that religiousness and a life of faith have developmental aspects; we can, therefore, view them as having an evolutionary dimension with a direction of growth, at times including the notion of suffering through the process (see Whitehead ^72^ and “process theology”). As such, religiousness and a life of faith are not opposed to the natural sciences’ concept of evolution.

According to Pannenberg, “At the end of the [19th] century and in the first half of the 20th, sadly, Christian churches and theologians could not recognize that the teaching of evolution offers an unprecedented possibility to theology in regard to the possibility of its relationship with modern science. The fight against Darwinism was one of the mistakes resulting in the most serious consequences throughout the history of theology’s relationship with the sciences.” ^73^ In a small way, our findings can support this position and shed light on the fact that some form of religious belief could indefatigably spread and survive despite the lack of significant and noticeable biological effects. If, depending on the depth of spiritual understanding, a religious experience could influence lymphocyte count, then it might contribute to the prevention of or recovery from disease, hence providing an evolutionary advantage (in the sense that the natural sciences use this term). However, from a theologian’s perspective, the goal of evolution is not survival or accommodation in the narrow sense of the word. Teilhard de Chardin wrote, “As early as in St. Paul and St. John we read that to create, to fulfill and to purify the world is, for God, to unify it by uniting it organically with himself.” Moreover, God is “from this point of vantage in the heart of [the] matter, assuming the control and leadership of what we now call evolution.” ^74^ He stated that “through human socialization, whose specific effect is to involute upon itself the whole bundle of reflexive scales and fibers of the earth, it is the very axis of the cosmic vortex of interiorization which is pursuing its course.” ^74^ Pannenberg often alluded to God’s appearance in world history in the manifestation of Jesus as an event that can be considered portent, anticipating the future. ^75^ In this sense, healings are of signal value because they hint at a precursory picture of a harmonious God-human relationship, including all its psychical and physical aspects. Pannenberg wrote, if, “[like Teilhard de Chardin,] we can consider life’s evolution as the process of the creation of life forms of increasing complexity and at the same time becoming increasingly introspective, then we can also state that in the succession of different forms of life by creatures, is expressed the increase in the shareholding of the divine spirit, of life’s spirit.” ^73^^,^^74^

Faith is an evolutionary advantage in the theological sense because evolution aims to increase the shareholding of the divine spirit. ^74^ This is the endpoint at which the results of our measurements converge.

## Data availability

### Underlying data

Figshare: Healing Study Beres *et al.* - Part 1: Psychological and Immunological Changes after meeting a Chaplain coupled with Biblical Readings among Hospitalized Patients, https://doi.org/10.6084/m9.figshare.16750384.v1. ^76^

Figshare: Healing Study Beres *et al.* - Part 2: fMRI Changes after meeting a Chaplain coupled with Biblical Readings and Recalling the Visits with Audiotapes among Hospitalized Patients, https://doi.org/10.6084/m9.figshare.16751851.v1. ^77^

### Extended data

Figshare: HEALING Study Beres *et al.* - F1000Research - extended data, https://doi.org/10.6084/m9.figshare.17029715.v3. ^78^

### Reporting guidelines

Figshare: CONSORT checklist for “Healing through faith: meeting a chaplain coupled with biblical readings could produce lymphocyte changes that correlate with brain activity (HEALING study)”, https://doi.org/10.6084/m9.figshare.17029715.v3. ^78^

Data are available under the terms of the Creative Commons Attribution 4.0 International license (CC-BY 4.0).
