# Supplementary material for: Healing through faith: Meeting a chaplain coupled with biblical readings could produce lymphocyte changes that correlate with brain activity (HEALING study)
Source: F1000Res. 2024 Oct 30;10:1295. Originally published 2021 Dec 20. [Version 5] doi: 10.12688/f1000research.74504.5 (PMC12149807; doi:10.12688/f1000research.74504.5)
Supplement: Peer review history

# Reviewer report: Reviewer response for version 5

**DOI:** 10.5256/f1000research.173734.r386606

**Published:** 2025-06-09

**Authors:** Kathy B Hoppe

**Affiliations:** 1 Oral Roberts University, Tulsa, USA

This article examines the impact of a religious intervention on patients served in acute medical care who received visits from a church chaplain and Bible reading together. In blood draws and, in a smaller group, fMRI scans were performed. There was no central immunological effect, but changes in lymphocytes were linked to changes in angular gyrus brain activity, which is involved in word recognition. The research emphasizes the individualized and subjective character of spiritual care in individuals and how chaplains provide the support people need. It also examines the relationship between spirituality, the immune system, and the brain and potential health benefits of spiritual experiences in addition to the need for future research in this area.

 While the researchers performed a sound study, there are several posits offered that may be overly confident or speculative. On page 15, the researchers state, "...remarkably, we did not obtain obvious false positive results." This might be overly confident considering the small sample size. This statement should acknowledge the possibility that Type I errors may still be present. In addition, on page 17, the following statement, "...the growing inconsistency in lymphocyte changes seemed to reveal a sense of fascination and admiration mixed with tones of fear," may be overly speculative based on immunological data alone. State this more tentatively. One final note is related to page 19, which states, "...could exert an influence on the patients' lymphocyte count and hence alter their recovery from the disease, depending on the depth at which the chaplain's core message is understood." This may infer a direct causal link between cognitive understanding and physiological changes. This may oversimplify the study's findings. 

 The article addresses a theological framework, which is helpful. However, it remains unclear how such ideology relates to the interpretation of scientific results. A point of clarification would be helpful. Finally, the final statement on page 20, "Faith is an evolutionary advantage..." is a broad leap from the results of this study, which showed one single brain area. Focus more on how theology may help interpret the observations.

 Otherwise, this study is remarkably interesting and further replication is encouraged.

Is the work clearly and accurately presented and does it cite the current literature?

Yes

If applicable, is the statistical analysis and its interpretation appropriate?

I cannot comment. A qualified statistician is required.

Are all the source data underlying the results available to ensure full reproducibility?

Yes

Is the study design appropriate and is the work technically sound?

Yes

Are the conclusions drawn adequately supported by the results?

Partly

Are sufficient details of methods and analysis provided to allow replication by others?

Yes

Reviewer Expertise:

trauma, secondary trauma, burnout, spirituality, theology

I confirm that I have read this submission and believe that I have an appropriate level of expertise to confirm that it is of an acceptable scientific standard, however I have significant reservations, as outlined above.

# Reviewer report: Reviewer response for version 5

**DOI:** 10.5256/f1000research.173734.r336511

**Published:** 2024-10-31

**Authors:** Philip R Baldwin

**Affiliations:** 1 Baylor College of Medicine, Houston, Texas, USA

The authors attended to all my earlier comments very graciously. I recommend the work for indexing.

Is the work clearly and accurately presented and does it cite the current literature?

Yes

If applicable, is the statistical analysis and its interpretation appropriate?

Partly

Are all the source data underlying the results available to ensure full reproducibility?

Yes

Is the study design appropriate and is the work technically sound?

Partly

Are the conclusions drawn adequately supported by the results?

Partly

Are sufficient details of methods and analysis provided to allow replication by others?

Yes

Reviewer Expertise:

Applied math, functional mri, structural biology

I confirm that I have read this submission and believe that I have an appropriate level of expertise to confirm that it is of an acceptable scientific standard.

# Reviewer report: Reviewer response for version 4

**DOI:** 10.5256/f1000research.135237.r318896

**Published:** 2024-09-27

**Authors:** Philip R Baldwin

**Affiliations:** 1 Baylor College of Medicine, Houston, Texas, USA

I think the work is important and I would like to encourage this line of inquiry as much as possible. I have been involved with a similar study

 Neural correlates of healing prayers, depression and traumatic memories: A preliminary study" from August 2016.

 I appreciate your feedback and understand the importance of adhering to rigorous scientific standards. I believe it would be beneficial to ensure that only significant results are quoted in the article. After a thorough revision to align with these guidelines, I would be happy to accept the changes.

 For example, there should be no sentence in the opening summary of results like:

 " **Results:** Lymphocyte counts increased more often after the more successful visits, but the immunological changes were not significant. "

 All such statements should be moved to the discussion. It is not what ordinarily scientists would consider a result.

Is the work clearly and accurately presented and does it cite the current literature?

Yes

If applicable, is the statistical analysis and its interpretation appropriate?

Partly

Are all the source data underlying the results available to ensure full reproducibility?

Yes

Is the study design appropriate and is the work technically sound?

Partly

Are the conclusions drawn adequately supported by the results?

Partly

Are sufficient details of methods and analysis provided to allow replication by others?

Yes

Reviewer Expertise:

Applied math, functional mri, structural biology

I confirm that I have read this submission and believe that I have an appropriate level of expertise to state that I do not consider it to be of an acceptable scientific standard, for reasons outlined above.

# Response

**Published:** 2024-10-24

**Authors:** András Béres

**Affiliations:** Kaposi Mór Teaching Hospital, Hungary

**Responses to Philip R. Baldwin (Reviewer#3)**

 Thank you Philip R. Baldwin for undertaking the review of this clinical study, the significant outcome of which is the correlation of the fMRI measurements (left BA39) with an immune parameter (lymphocyte count).  

 We are honored to now have a psycho-endocrinologist and two neuro-researchers review our article regarding the impact of discoveries in psycho-neuro-immunology (PNI) in clinical practice. Additionally, we are especially pleased to learn that the reviewers reflect the diverse representation of gender and geographic location that is advocated for by the F1000Research open publishing platform hosting this article.  

 Please find below our responses to your comments.

 “I believe it would be beneficial to ensure that only significant results are quoted in the article. After a thorough revision to align with these guidelines, I would be happy to accept the changes. 

 For example, there should be no sentence in the opening summary of results like the following: ‘Results: Lymphocyte counts increased more often after the more successful visits, but the immunological changes were not significant.’ 

 All such statements should be moved to the discussion. This is not what scientists would ordinarily consider to be a result.” 

 Response: We are particularly pleased that you considered the presentation of the literature to be well-founded, the supporting data to be credible, and the presentation of the method to be reproducible. We trust that thanks to our detailed corrections, you will now consider the interpretation and conclusions to be acceptable. 

 The non-significant findings are important from the standpoint of estimating the extent of neuro-immunomodulation in a clinical setting, but we have moved these results (and especially emerging trends) to the Discussion. The trends may be interesting to the reader because they resonate with previous studies (suggesting that the sample size of our study was underpowered for the measurement of immune changes), but the most important finding is that in a clinical environment, the possibilities of inducing psycho-immunological effects become narrower. 

 At the same time, the fMRI-immune correlation gives us hope that even in this situation, burdened with limitations, the mechanisms that can be relied upon for recovery will remain.

# Reviewer report: Reviewer response for version 4

**DOI:** 10.5256/f1000research.135237.r322888

**Published:** 2024-09-12

**Authors:** Vaitsa Giannouli

**Affiliations:** 1 Department of Psychology, University of Western Macedonia & School of Medicine, Aristotle University of Thessaloniki, Thessaloniki, Greece

This interesting article presents findings of a research on the immunological and neurological changes in hospitalized patients after meeting a chaplain coupled with biblical readings. One of the points that authors need to justify in the Methodology is the sample size. How was it estimated? The generalizability of the findings is under question due the small sample size of the three groups. In the Introduction (first paragraph) authors do not need to report statistics from other studies. In addition, in the Introduction authors need to add a separate section focusing on religion and spirituality as therapeutic factors in older patients and also discuss their role in different cultural contexts (e.g. for a relevant review which explains these two different concepts: http://www.encephalos.gr/pdf/58-2-02e.pdf). For relevant articles on the positive effects of these variables authors can find information in the following recently published articles (doi: 10.1007/978-3-030-32637-1_8

 doi: 10.1007/978-3-031-31986-0_35). Please provide more details regarding the intervention (so researchers can replicate this study). Please describe in more detail the recruitment procedure. Please describe the figures in the main text as they are difficult to follow. One more point to consider for a major revision is the structure of the Discussion (please avoid starting with limitations), but present first the main findings and clinical applications and then the limitations.

Is the work clearly and accurately presented and does it cite the current literature?

Partly

If applicable, is the statistical analysis and its interpretation appropriate?

Partly

Are all the source data underlying the results available to ensure full reproducibility?

Partly

Is the study design appropriate and is the work technically sound?

Partly

Are the conclusions drawn adequately supported by the results?

Partly

Are sufficient details of methods and analysis provided to allow replication by others?

Partly

Reviewer Expertise:

Psychology and endocrine diseases

I confirm that I have read this submission and believe that I have an appropriate level of expertise to confirm that it is of an acceptable scientific standard, however I have significant reservations, as outlined above.

## References

[1] : Subjective Quality of Life, Religiousness, and Spiritual Experience in Greek Orthodox Christians: Data from Healthy Aging and Patients with Cardiovascular Disease. *Adv Exp Med Biol* .2020;1196: 10.1007/978-3-030-32637-1_8 85-91 10.1007/978-3-030-32637-1_8 32468309

[2] : Better Understand to Better Predict Subjective Well-Being Among Older Greeks in COVID-19 Era: Depression, Anxiety, Attitudes Towards eHealth, Religiousness, Spiritual Experience, and Cognition. *Adv Exp Med Biol* .2023;1425: 10.1007/978-3-031-31986-0_35 359-364 10.1007/978-3-031-31986-0_35 37581810

# Response

**Published:** 2024-10-24

**Authors:** András Béres

**Affiliations:** Kaposi Mór Teaching Hospital, Hungary

**Response to** **Vaitsa Giannouli (Reviewer#2)**

 Thank you, Vaitsa Giannouli for joining the open peer review process for our article.

 We are honored to now have a psycho-endocrinologist and two neuro-researchers review our article regarding the impact of discoveries in psycho-neuro-immunology (PNI) in clinical practice. Additionally, we are especially pleased to learn that the reviewers reflect the diverse representation of gender and geographic location that is advocated for by the F1000Research open publishing platform hosting this article.

 Please find below our detailed responses to your comments.

 “One of the points that authors need to justify in the Methodology is the sample size. How was it estimated? The generalizability of the findings is under question due the small sample size of the three groups.” 

**Response:** We have now clearly indicated in the Introduction that the number of cases was principally limited by financial constraints. We were forced to limit our goals to the number of cases reaching that of the pilot studies conducted with healthy participants, which is addressed in the Introduction. 

 We have also outlined the scope of conclusions that can realistically be drawn from the test results. Whether the observed changes would have been significant in relation to the same immune parameters with a larger number of cases remains an open question; however, owing to the same number of cases as in previous research, we can establish that compared to studies conducted with healthy participants, the magnitude and possibility of neuro-immunomodulation in the clinic is more limited.

 “In the Introduction (first paragraph) authors do not need to report statistics from other studies.”  

**Response: **Based on the above, we consider it important to retain the brief references to the sample sizes of the pilot studies that we mention in the Introduction.  

 As for the p-values, we consider it important to mention them as we now emphasize in the Introduction that influencing the immune system with psychological factors is already difficult, even in the case of healthy volunteers (as shown by several p-values ​​close to the limit value).

 “In addition, in the Introduction, authors need to add a separate section focusing on religion and spirituality as therapeutic factors in older patients and also discuss their role in different cultural contexts.”  

**Response:** We have now amended the Introduction with a reference to religion and spirituality as therapeutic factors affecting health.  

 It was interesting to read that quality of life-life satisfaction, religiosity, and spirituality were not strongly interconnected in your Greek Orthodox Christian sample (DOI: 10.1007/978-3-030-32637-1_8); this underscores how difficult it is to truly enforce the confirmed positive effects of spirituality on the entire body in the whirlwind of real life beyond clichés. Our study shows the same in the immersive experience of a hospital setting. 

 This is also why we consider it appropriate and forward-thinking to incorporate theology into science by investigating the relationship between religion and health (as outlined in the expanded excursus: DOI: 10.1007/s10943-023-01888-3). Theology paves the way for understanding measured outcomes by demonstrating how the religious approach reinterprets the overall concept of health, which ultimately affects the choices and lifestyles of religious people. Christian theology is a striking example of this, where choosing a fuller life also implies taking up the cross, sometimes prompting choices that are in stark contrast to Western ideas of comfort.

 “Please provide more details regarding the intervention (so researchers can replicate this study).”  

**Response: **We have now explained the intervention in more detail under "Intervention and measurements."

 “Please describe in more detail the recruitment procedure.” 

**Response:**  We have now explained the recruitment procedure in more detail under "Enrollments,” with particular regard to the more detailed description of the HEAL Score. The HEAL Score template form can be downloaded from the Extended data (Appendix S1), as indicated in the article. 

 According to our approach, the religious experience, due to its nature, cannot be examined in precisely the same way as other psychological experiences; even the elicitability of the experience and its measurability are questionable. If we assume that a transcendent spiritual experience can be considered an encounter between the individual and the transcendent Other, then the scientific investigation of this experience also has to take into account that the experience may be understood as both an inter-personal and intra-personal event. 

 In this approach, the research design of natural scientific inquiry into a spiritual experience should focus on preserving the authentic nature of the encounter as much as possible.  When examining a religious experience, we consider it essential to ensure authenticity, personalization, and spontaneity, which we now further emphasize in the Discussion. 

 “Please describe the figures in the main text as they are difficult to follow.”  

**Response: **Thank you for pointing this out; we have now included an explanation of the figures in the text.

 “One more point to consider for a major revision is the structure of the Discussion (please avoid starting with limitations), but present first the main findings and clinical applications and then the limitations.” 

**Response:** Following Reviewer#3’s suggestion, the revised version focuses on the study’s more significant results; we have placed the non-significant results and limitations at the end of the Discussion.

 We have concluded by addressing the issue of clinical applicability in order to summarize the overall picture emerging from the pattern of significant and non-significant findings: In a clinical setting, the possibilities of neuro-immunomodulation are narrower.

# Reviewer report: Reviewer response for version 1

**DOI:** 10.5256/f1000research.78267.r120035

**Published:** 2022-02-21

**Authors:** Andrew B. Newberg

**Affiliations:** 1 Department of Integrative Medicine and Nutritional Sciences, Marcus Institute of Integrative Health, Thomas Jefferson University, Philadelphia, PA, USA

Regarding the article entitled, “Healing through faith: meeting a chaplain coupled with biblical readings could produce lymphocyte changes that correlate with brain activity (HEALING study)”, overall, this study is potentially quite interesting and would be of interest. However, the lack of positive results and the confusing presentation with heavy reliance on religious and theological discussion substantially limits the value of this article from a scientific perspective.

**Introduction**

 The introduction needs to clearly state what the authors’ hypotheses were. Without this, it is difficult to understand what they are trying to accomplish in their study. Do they think that lymphocyte counts will increase or decrease? What correlations are they expecting? What parts of the brain are they anticipating will be involved.

 They should consider elaborating on studies of mindfulness and other meditation practices on immune function.

**Methods**

 The authors should clarify how subjects were allocated since they were not truly randomized, but allocated by convenience.

*The authors state:* *The decision was based on the patient’s degree of need and willingness, as assessed by, and at the discretion of, the chaplain…*

 What does this sentence mean? Is the chaplain deciding who is included in the study? This seems highly problematic.

*The authors state: The randomized control group consisted of patients who knew the goal of the measurement but were explicitly asked to help with their participating in the control group, i.e., they knew they weren’t going to meet the chaplain (by request, the encounter could be scheduled for a later occasion).* Does this mean that they knew they were controls or just that they weren’t meeting with the chaplain?

 The authors state blood collections occurred 30 minutes prior to and 60 minutes after the spiritual intervention. Can they provide an explanation as to why this particular timing? Is this based upon any data regarding the speed with which these serum markers can change?

 Regarding the passages read to the subjects, is it possible that the Isiah chapter is something that the participants know well? Would it have been better to have read from some other passage of the bible such as a listing of names which has a potential neutral effect rather than something that is medically oriented?

**Part I Results**

 This comes across as a very confusing section. The tables and figures are very confusing and in the end, it seems that no results were significant. Perhaps this section can be substantially altered to state that there were no significant findings. If there was a trend towards significance in any of the values, perhaps the authors can use this information to provide an effect size that might be useful for developing future studies. But otherwise, it does not seem appropriate to include tables and complex figures on data that is non-significant.

**Part II Results:**

 Perhaps this was not clear, but were there differences between the active prayer block and the control block or just between prayer and silence? This might be particularly relevant and interesting in that hearing a religious recitation affects the brain differently than a neutral/scientific recitation. This might also be more relevant in terms of other physiological and psychospiritual measures. Otherwise, as with the section above, the authors should really only show the significant results and can delete Figure 11.

 In the text, the authors state: *After that, we aimed to correlate the dLy parameters with the areas showing change in the fMRI activity. An invert linear correlation emerged (p = 0.019 with dLy%, p = 0.003 with dLy-abs) between the change in activity of the left BA39 area and the change in lymphocyte counts*

 Can they clarify that this refers to the active minus silence condition? Again though, perhaps it would be more interesting to see correlations between the active and scientific control?

**Discussion:**

 The first few sentences of the Discussion do not seem to be relevant to the main discussion and should be deleted:

*Taking Salopek’s “slow journalism” concept as a model [he makes a 21,000 mile-long journey (33.780 km) by foot, to retrace the pathways of our ancestors, the first humans who migrated from Africa and journeyed around the Earth - he posits that one must devote time to inter-personal connections in order to fully comprehend the human phenomenon - “Out of Eden Walk,”* *^35^* *], we consciously stood for the concept of “slow science” for a slow pace of methodology.*

 In the Discussion, the authors refer to the auto-control setting. Can the authors clarify what they mean by this?

 It is not clear why the SHoRT trial is being used for comparison since this was a study of children. It seems that the design is completely different as well focusing on artistic components rather than spiritual. This should either be deleted or expanded more to clarify why this is an appropriate study for comparison.

 The authors state that the intervention was designed to elicit positive emotions, but may have resulted in other emotions as well. The authors should review articles on positive emotions and immune function. In addition, there should be greater clarification of what other emotions were elicited. Did they acquire data for this?

 Can the authors clarify the following passage? What do they mean by a “guiding principle”?

*however, the correlation of these changes with the fMRI-s indicated that even when biblical readings did not appear to have a physiological effect, they could have an ordering, arranging effect, along some specific guiding principle, on a key parameter like the lymphocyte counts.*

 It is appreciated that the authors try to connect the angular gyrus to spiritual aspects of the experience, but this needs to be clarified in the Introduction as well. On the other hand, the authors state: *Thus, the brain area involved in the correlation described above (gyrus angularis) could also be linked to the “Aha! moment”.* Why is there suddenly discussion about an Aha moment when this has not been discussed before and there is no mention of it in the methods or results? This should probably be deleted.

 Can the authors clarify why there were few significant findings? Perhaps it would be better to discuss the meaning of the lack of significant findings.

 The entire last part of the Discussion beginning with the paragraph that starts: “When one is examining the effect of the Word of God on man,…” seems to be speculation, theology, and not based on the scientific results presented. This whole section should be deleted.

 While the goal of the authors is appreciated, it seems that the limitations of the article described above are substantial.

Is the work clearly and accurately presented and does it cite the current literature?

Partly

If applicable, is the statistical analysis and its interpretation appropriate?

No

Are all the source data underlying the results available to ensure full reproducibility?

Partly

Is the study design appropriate and is the work technically sound?

Partly

Are the conclusions drawn adequately supported by the results?

No

Are sufficient details of methods and analysis provided to allow replication by others?

Partly

Reviewer Expertise:

Neuroimaging, meditation

I confirm that I have read this submission and believe that I have an appropriate level of expertise to state that I do not consider it to be of an acceptable scientific standard, for reasons outlined above.

# Response

**Published:** 2022-02-21

**Authors:** András Béres

**Affiliations:** Kaposi Mór Teaching Hospital, Hungary

First of all, thank you Andrew Newberg for accepting our invitation and becoming the first to openly peer-review this paper. Thank you for going through the article so thoroughly with us.  

 This is to confirm that yes, it is the correlation between change in lymphocytes, and difference in activation between the active and the scientific control block that was significant (not between the active and the silence block; the abbreviation "s" refers to the "scientific text" 's block).

 This is also to clarify that main purpose of the study was to measure the potential immunological effects of personal interventions that are primarily intended to elicit positive emotions at the bedside, among patients lying in the hospital. In the everyday practice of a hospital, this practically translates to visits by artists for the child-, and by chaplains for the adult population. This explains why we can refer to these two, seemingly different interventions on the same page in this study. "SHoRT" Study examined the effect of artists among sick children, "HEALING" Study examined the effect of a chaplain among sick adults.   

 The methodological challenge was that genuine meetings with chaplains in hospitals always contain a spontaneous ("inspired") element, the very creation of such authentic meetings is always based on the chaplain's intuition, and this spontaneous element has a major effect on the whole meeting - and hence on any immunological imprint, would there be any. This spontaneity and the large room left for the chaplain's inspiration reflects the original setting of the biblical times in which the "Word of God" was firstly said, when such meetings happened as a spontaneous act of the participants - i.e., this is how Jesus met people. Had we taken this spontaneous element out, we would have suffocated the very phenomenon of which we were trying to estimate the potential immunological imprint. 

 This methodological decision to leave the spontaneous element in, does not affect the reproducibility of the study - to the contrary, by proposing the possibility of the meeting for EVERY patient available on a ward on the day of the measurement, by establishing a rough order for the visits ("HEAL Score") to help manage the time available, but by always leaving to the chaplain the final choice of the patient she intends to visit, and only asking the chaplain to include the same biblical passage in every spontaneous meeting: these are the ways this study proposes to be reproducible anytime and anywhere in the world. 

 The question raised was whether meetings with a chaplain can elicit immunological changes in patients, IF these meetings are left as authentical, as spontaneous as they originally are.      

 It is the determination of a concrete group of patients among which to choose, the instruction for the chaplain to remain spontaneous and the permanence of the concrete biblical passage read that are fixed. 

 In contrast to the previous studies on praying and meditation, the main phenomenon that this study was focused on was the effect of a spiritual meeting - a meeting with the chaplain and through her, with the "Word of God". Thus, the phenomenon in focus in this study was not praying, but meeting. 

 It is the religious sensibilities raised by the topic touched by this measurement, and to avoid any potential distorsions in the interpretation of the results that we found, that we felt obliged to include the theological viewpoint in the discussion of the results. I.e. the reader - believer or atheist - could later ask: WHY have we not measured more immunological changes, if the "Word of God" is believed to heal? And, if lymphocyte counts are correlated with an area of the brain involved in understanding words, does that mean that those who TRY to understand the biblical passage better, will have more healing lymphocytes? These are theological questions.         

 Thank you for being with us on this journey.  

 We hope that these clarifications will prove to be meaningful for you. We believe these are also the main topics the readers will possibly face while summing the article up in themselves. 

 Based on the Editorial Team's suggestion, we will be waiting for additional peer review reports before starting on the article revisions, in which we will address all the issues raised in full detail. We hope these improvements will render the article acceptable for you.

# Response

**Published:** 2022-03-17

**Authors:** András Béres

**Affiliations:** Kaposi Mór Teaching Hospital, Hungary

We are now publishing a revised version (v3) of our article, in order to address your report in full detail. 

 To this we would add that the Isaiah chapter was expected to be less known by the participants, since it is seldom quoted and most of the enrolled patients were not actively practicing their faith; the proposition raised in your report, to use a different control as neutral, and to read from some other passage of the Bible such as a listing of names, is very interesting.

 A potentially surprising twist to this idea is that such a list of names, as neutral as it seems to be from a grammatical point of view, is not neutral from a spiritual point of view. Indeed, central in Judaism and in Christian piety is the assumption that the *whole* Bible - and not just parts of it - is sacred, the emotionally exuberant and the less spectacular verses alike. Interestingly, then, even the listing of names could hold a deeper meaning in this context: in line with Hellinger's theory stating that our "self" cannot be conceived as an individual being, but our subconscious is deeply rooted in the soul of our family, that of our ancestors, in the wider sense of the whole family of mankind (1, 2), the recitation of ancient names is portent of deep memories, thus can be evocative, and has the potential to move the deepest layers of the collective subconscious.  

 The interesting question is whether these potential effects can be measured with fMRI, and whether this additional spiritual content would only be perceived by the religious participants, or some unintended imprints would also be detectable among the atheists. What do you think? 

 In the current measurement, the usage of a text without any spiritual content as control seemed more appropriate, to maximally differentiate it from the biblical reading used in the active block.

 The comparison of biblical passages with different emotional content during fMRI, as well as the more sensitive measurement of the immunological parameters (by means of flow-cytometry for example, a tool that we could not afford due to financial constraints) are lines along which further research could develop - but this already paves the way for a series of completely new measurements.    

 References

 (1) Hellinger B: *Rachel Weeping for Her Children: Family Constellations in Israel.* Heidelberg, Germany: Carl-Auer-International; 2003.

 (2) Hellinger B: *Peace begins in the soul: Family constellations in the service of reconciliation *(C. Beaumont, Trans.). Heidelberg, Germany: Carl-Auer-Systeme Verlag; 2003.

# Response

**Published:** 2022-06-17

**Authors:** András Béres

**Affiliations:** Kaposi Mór Teaching Hospital, Hungary

Dear Andrew B. Newberg,

 We are now publishing a further revised version of the article "Healing through faith: meeting a chaplain coupled with biblical readings could produce lymphocyte changes that correlate with brain activity (HEALING study)”, in order to fully address the concerns you articulated regarding Version1.

**Introduction**

 Based on your advice, we have now clarified what our hypotheses were, what we were expecting to find in regards of changes in the immune response and the involvement of which brain areas could be anticipated. The truth is we intentionally started this clinical trial with as few preconceptions as possible, to remain open to any significant correlation that could emerge between the parameters we could afford to measure in a clinical setting – some of which are traditionally considered to correspond to the body, some others to the soul. Our measurement is a pilot study in this respect.  

**Methods**

 We have further clarified why the subjects were not randomized by chance, but by convenience i.e., depending on the availability of the chaplain on the days of the measurement (when the laboratory staff was available for the extra measurements and when the fMRI equipment could be used for the study). Allocating by chance would not have been a difficult option technically but could have resulted in significant distortions in the supposed psycho-immunological effects.  Indeed, by the time the patients were classified as eligible and randomizable in this study, they represented this tiny portion of patients who were truly in need of a visit by the chaplain. The cancellation/postponement of the visit could have resulted in significant stress, disappointments, by which means the measurement method would have disproportionately influenced the parameters measured in the control group. 

 In the intervention group, it was the chaplain who decided whom she wanted to visit among the patients who were randomizable. In the Discussion, we explain in detail why we opted for this methodological decision – the main goal was not to interfere with the genuine, authentic character of the chaplain-patient relationship that we were aiming to understand, and that always contains such a spontaneous element.   

 The timing of the blood collections (30 minutes prior, and 60 minutes after the meeting) is now explained, it was based on the similar intervals used by previous, basic psycho-immunological studies that served as our starting point and that are mentioned in the Introduction (especially Berk et al., Bennett et al., Matsuzaki et al.). 

 We believe the Isaiah chapter we used is, although not an entirely neglected, but a rarely used passage in religious practice, moreover, most of the patients enrolled were not actively practicing their faith. As to the usage of another biblical passage such as a listing of names for neutral control, we refer you back to our response in Version3 – even “neutral” passages can be portent of a “numinous” component. The contrast between a sacred passage intended to offer comfort, and a medical text with potentially fearful connotations was more marked, and therefore helped bring out the potential differences better.     

**Part I Results**

 Based on your suggestions, we have now significantly simplified this section and moved all the figures and tables containing null-results to the *Extended data*, so as not to overwhelm the reader with data that is non-significant. Also based on your suggestions we added data on effect size when there seemed to be a trend towards significance that might help developing further studies. 

**Part II Results**

 We have unambiguously clarified the meaning of the “a”, “s” and “c” abbreviations.

 There were no general differences that could be unanimously detected among all the patients, between the active prayer block and the control block, so hearing a religious recitation did not affect the brain differently than a neutral/scientific recitation, unanimously for all the patients enrolled in this study – but the main finding of the study is that a significant correlation between lymphocytes and brain activity emerged with the active minus scientific control block. This finding totally echoed previous psycho-immunological findings and is now highlighted in the Discussion.

 We hope your assessment of the statistical analysis being not appropriate was related to the confusing presentation of the null results, and that this issue is now fixed. The statistical tools used for interpretation, especially for fMRI evaluation are detailed in the Methods section and the Results part now is now limited to the precise description of the positive findings with some careful allusion to possible trends. We added the r value for the main correlation found.    

**Discussion**

 We left the reference to Salopek’s *“Out of Eden Walk”* in the Discussion but put it to the end of the first part, to show that this reference is meant to clarify the methodological decisions of the authors – a constant effort to produce authentic encounters at the bedside or in the chapel, even at the cost of a very slow pace of enrollment. We further illustrate this concept with the example of two patients whom we enrolled by proactively trying to overcome the special difficulties raised by their disabilities. Only a very personified study design could allow for this.

 Also, the term” auto-control” setting is now clarified in the text.

 It is particularly important that patients’ individual results are first compared to their own baseline values (difference in their own activation patterns during intervention and silence; difference in their own lymphocyte counts), in the context of a study using a cohort design, with many different patients enrolled.    

 The SHoRT trial is used for comparison in the Discussion because either the artistic interventions for hospitalized children or the spiritual interventions for hospitalized adults were originally implemented in clinical practice with the implicit intent to elicit positive emotions among patients, to alleviate the burden caused by the disease. This is now explained in the Introduction, and based on your suggestion, the revised Introduction has been considerably enriched with additional key articles on positive emotions and immune function. These additional references help understand why the current study needed to put so much emphasis on proper patient selection, authenticity of the interventions; they also put the current study’s sample size, measured immune parameters, obtained p values, direction of change (increase or decrease) in perspective, as compared with similar basic studies.

 The revised Introduction also points towards a more nuanced approach to the visits by a person of faith, with a highlight on the religious undertones, and the revised Discussion also elaborates on that the intervention originally designed to elicit positive emotions, may have resulted in other emotions as well. The lymphocyte changes in the subgroup who received communion gives a striking example. The „Healing questionnaire” designed to record rough psychological aspects of the meetings, like emotional depth, perceived turmoil, correspondence with the chaplain as assessed by the participants… shows lines for further research, but the amount and length of questionnaires needed to be considerably restrained in the current setting, so as not to overwhelm the patients with documents to be completed - that could have interfered with the meetings themselves. As the Discussion acknowledges, the different emotional aspects of the spiritual experience in hospitals will need further research.            

 The concept of a “guiding principle” having an arranging effect on the lymphocyte counts is now clarified in the Discussion.

 Based on your suggestion, we clarified in the Introduction how the angular gyrus could be involved in the spiritual experience, so that its appearance in the Discussion does not come across as a totally unexpected finding. Also based on your suggestion we deleted the allusion to the ‘Aha moment’ from the Discussion (correlations are possible but seem too remote to be dealt in detail in the Discussion of this study). 

 Finally, although we keep the final part of the Discussion for the theological perspectives, we’d like to highlight that this section is called “Excursus”. We think it is always interesting to see how empiric observations relate to the way theology thinks about God. This is not meant as a substitute to replace the scientific discussion, but a way to complement it.   

 We hope that with these significant revisions in the Discussion, your assessment regarding the conclusions as not having been drawn as adequately supported by the results is now changed.

 We are honored by your accepting to review this study. We honestly appreciate your reviewing it so thoroughly and hope our revisions have sufficiently addressed your concerns so that you now find the article acceptable from a scientific perspective.
